# Coalescent Analysis of Phylogenomic Data Confidently Resolves the Species Relationships in the *Anopheles gambiae* Species Complex

**DOI:** 10.1093/molbev/msy158

**Published:** 2018-08-09

**Authors:** Yuttapong Thawornwattana, Daniel Dalquen, Ziheng Yang

**Affiliations:** 1 Department of Genetics, Evolution and Environment, University College London, London, United Kingdom; 2 Department of Microbiology, Faculty of Science, Mahidol University, Bangkok, Thailand; 3 Radcliffe Institute for Advanced Studies, Harvard University, Cambridge, MA

**Keywords:** *Anopheles*, bpp, coalescent, introgression, inversion, species tree

## Abstract

Deep coalescence and introgression make it challenging to infer phylogenetic relationships among closely related species that arose through radiative speciation events. Despite numerous phylogenetic analyses and the availability of whole genomes, the phylogeny in the *Anopheles gambiae* species complex has not been confidently resolved. Here we extract over 80, 000 coding and noncoding short segments (called loci) from the genomes of six members of the species complex and use a Bayesian method under the multispecies coalescent model to infer the species tree, which takes into account genealogical heterogeneity across the genome and uncertainty in the gene trees. We obtained a robust estimate of the species tree from the distal region of the X chromosome: (*A. merus*, ((*A. melas*, (*A. arabiensis*, *A. quadriannulatus*)), (*A. gambiae*, *A. coluzzii*))), with *A. merus* to be the earliest branching species. This species tree agrees with the chromosome inversion phylogeny and provides a parsimonious interpretation of inversion and introgression events. Simulation informed by the real data suggest that the coalescent approach is reliable while the sliding-window analysis used in a previous phylogenomic study generates artifactual species trees. Likelihood ratio test of gene flow revealed strong evidence of autosomal introgression from *A. arabiensis* into *A. gambiae* (at the average rate of ∼0.2 migrants per generation), but not in the opposite direction, and introgression of the 3 L chromosomal region from *A. merus* into *A. quadriannulatus*. Our results highlight the importance of accommodating incomplete lineage sorting and introgression in phylogenomic analyses of species that arose through recent radiative speciation events.

## Introduction

The *Anopheles gambiae* species complex is a group of sub-Saharan African mosquito species that is comprised of at least eight recognized species and includes major malaria vectors in Africa. These species are morphologically nearly indistinguishable but genetically distinct, and have different ecological traits and reproductive behaviors (such as range, habitats, resting and feeding preferences, and vectorial capacity) (Coluzzi et al. 1979; White et al. 2011). Three members, *A. gambiae*, *A. coluzzii*, and *A. arabiensis*, are ecologically most similar, with large overlapping geographical ranges across sub-Saharan Africa and are major malaria vectors (Wiebe et al. 2017). *Anopheles gambiae* and *A. coluzzii* are closely related sibling species that are highly anthropophilic and are responsible for the majority of malaria transmission in Africa, whereas *A. arabiensis* is a less dominant vector (Takken and Verhulst 2013). *Anopheles melas* (or its sister species *A. bwambae*) and *A. merus* are salt-tolerant species that breed in brackish coastal waters of eastern and western Africa, respectively. They have similar ecological and morphological characteristics and are minor vectors (Coluzzi et al. 1979). *Anopheles quadriannulatus* and its sister species *A. amharicus* bite animals and play no role in malaria transmission despite vector competence for *Plasmodium falciparum* (Sinka et al. 2012; Wiebe et al. 2017).

Inference of evolutionary relationships among those species is fundamental to identifying genomic changes associated with epidemiologically important traits and for developing effective malaria control strategies. Knowledge of the species phylogeny is also important to genome sequence assembly, inference of genome rearrangements, and reconstruction of ancestral genomes (Anselmetti et al. 2018). However, phylogenetic resolution of the *A. gambiae* species complex has been extremely challenging. First, the rapid succession of speciation events in the species complex combined with large population sizes of the ancestral species has caused widespread genealogical heterogeneity across the genome or incomplete lineage sorting (ILS) (Ayala and Coluzzi 2005; Fontaine et al. 2015). Second, introgression is prevalent in autosomal regions of the genome, in particular involving the three major vector species *A. gambiae*, *A. coluzzii*, and *A. arabiensis* (Besansky et al. 2003; Wang-Sattler et al. 2007; O’Loughlin et al. 2014). Third, different genomic regions, such as the X chromosome, the autosomes, and the inversion regions on chromosomes 2L and 3L, show systematically different phylogenetic relationships, possibly due to complex effects of chromosomal inversion, introgression, and natural selection (Slotman et al. 2005; Ayala et al. 2017). Inversions (both fixed and polymorphic) are prevalent across the genome (Coluzzi et al. 2002) and are associated with adaptation in different ecological habitats (Ayala et al. 2017). As a result of those complicating factors, different types of data support different species phylogenies for the species complex. *Anopheles arabiensis* and *A. gambiae* + *A. coluzzii* are ecologically most similar. Their close relationship is supported by sequence data in the autosomal regions, the Y chromosome (Hall et al. 2016) and the mitochondrial genome (Fontaine et al. 2015), but not by chromosomal inversions (Coluzzi et al. 1979, 2002) or the X chromosome (Fontaine et al. 2015). Similarly ecology and morphology groups *A. merus* with *A. melas*, but this sister relationship is contradicted by genetic data including genomic sequences and chromosomal inversions (Coluzzi et al. 1979, 2002).

Fontaine et al. (2015) provided the first phylogenomic analysis of the species complex using complete nuclear and mitochondrial genomes of six members: *A. gambiae*, *A. coluzzii*, *A. arabiensis*, *A. merus*, *A. melas*, and *A. quadriannulatus*. The maximum likelihood (ML) phylogenies from 50-kb nonoverlapping windows sliding along the genome showed widespread cross-genome heterogeneity in genealogical history. The X chromosome and autosomes produced drastically different phylogenies. The authors provided evidence that the majority tree for the X chromosome represents the true species branching order, whereas extensive introgressions have altered the autosomal phylogeny. However, the sliding-window (concatenation) approach used in Fontaine et al. (2015) fits one tree to all sites in the large window and ignores the coalescent process in the ancestors or the ILS. For closely related species formed through radiative speciations, concatenation is well-known to be unreliable (Edwards et al. 2016): it may be inconsistent and converge to a wrong species tree when the amount of data increases (Kubatko and Degnan 2007; Roch and Steel 2015). The same genomic data were analyzed using a phylogenetic network model with coalescent that captures both ILS and gene flow between species (Wen, Yu, Hahn, et al. 2016; Wen, Yu, and Nakhleh 2016), producing different phylogenies from those of Fontaine et al. (2015). Nevertheless, this analysis treated inferred gene trees as input data and ignored information in gene-tree branch lengths. As a result, they may lack power and fail to account for uncertainties in the gene trees due to limited phylogenetic information at each locus (Xu and Yang 2016).

We compile data sets consisting of loosely linked short genomic segments (called “loci”) from the genomes of six members of the *A. gambiae* species complex (Fontaine et al. 2015) and perform Bayesian species tree analysis using the program bpp (Yang 2015). Bpp implements the multispecies coalescent (MSC) model (Rannala and Yang 2003, 2017; Yang and Rannala 2014), and explicitly accommodates gene-tree heterogeneity across loci and makes a full use of information in the data including information in coalescent times or branch lengths while accommodating the uncertainties in the gene trees. We compile and analyze separate data sets for the coding and noncoding regions of the genome. We also perform concatenation analysis using RAxML (Stamatakis 2014) on these data sets, mimicking the sliding-window analysis of Fontaine et al. (2015). We use simulation informed by the real data to understand the different species tree estimates produced by the coalescent and concatenation analyses. As bpp does not account for gene flow between species, we use the ML program 3s to test for migration and estimate migration rates (Zhu and Yang 2012; Dalquen et al. 2017). While limited to only three species, this is a full likelihood implementation of the isolation-with-migration (IM) model (Hey and Nielsen 2004) and allows gene flow between two ingroup species with a third species used as outgroup. Our analysis leads to a robust species phylogeny for the *A. gambiae* species complex, providing a framework for studying the evolution of ecological and epidemiological traits. As an example, we discuss the implication of our species tree on the evolution of the 2La inversion polymorphism, which is an important epidemiological trait associated with susceptibility to *Plasmodium* infection in natural mosquito populations (Riehle et al. 2017).

## Results

### Species Branching Order Varies Systematically Among Different Parts of the Genome

We compiled 57,592 noncoding and 23,505 coding loci using the whole genome alignments of Fontaine et al. (2015) for 6 species in the *A. gambiae* species complex: *A. gambiae* (G), *A. coluzzii* (C), *A. arabiensis* (A), *A. melas* (L), *A. merus* (R), and *A. quadriannulatus* (Q). The loci are 100–1,000 bases in length and are at least 2 kb apart. Our analysis assumes the molecular clock so that rooted trees can be inferred without the outgroup but we also constructed data sets that include *A. christyi* (O) as the outgroup. The genome is partitioned into ten chromosomal regions (supplementary table S1, Supplementary Material online). For computational tractability of bpp and to explore the heterogeneity in species relationships across the genome, we split each data set into blocks of 100 loci, so that there are 582 noncoding blocks and 238 coding blocks. Each block is analyzed using bpp to calculate the posterior probabilities for species trees (the A01 analysis in Yang 2015).

**Fig. 1.**
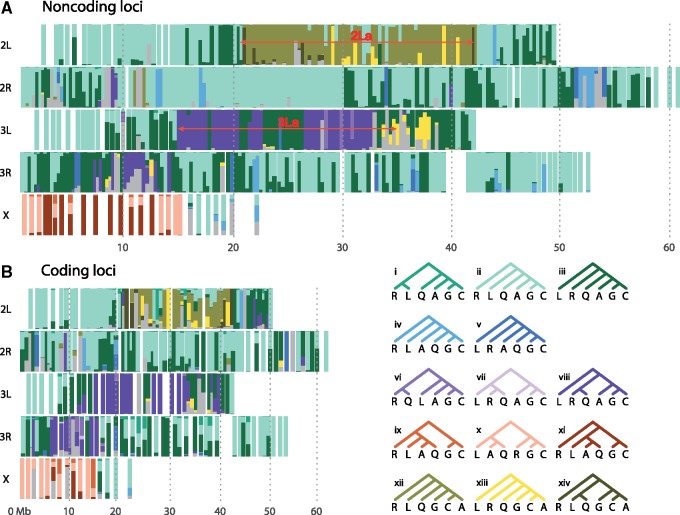
Posterior probabilities of species trees inferred using bpp for 100-locus blocks of (*A*) noncoding and (*B*) coding loci. The *y*-axis scales from 0 to 1. The *x*-axis provides approximate chromosomal coordinates of blocks, where the position for each block was taken to be the average of the starting positions in AgamP3 coordinates over all loci within the block.

**Fig. 2.**
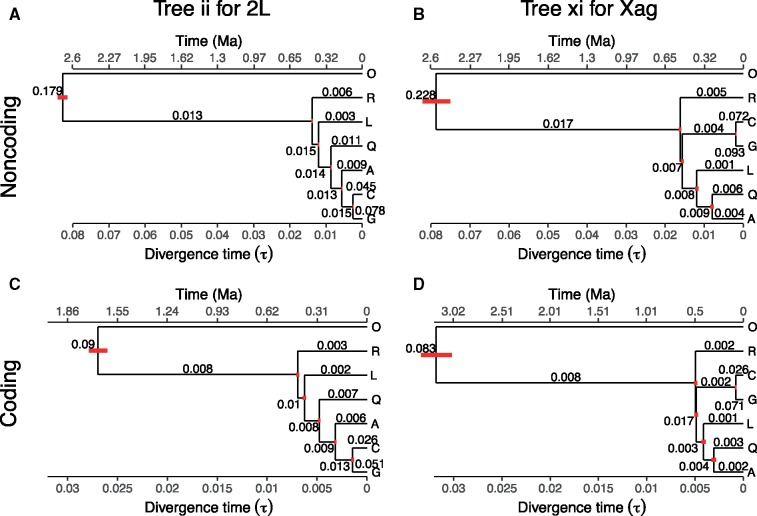
Trees ii and xi with posterior estimates of population sizes (*θ*s, numbers on the branches) and species divergence times (*τ*s, the bottom horizontal axis; bars represent 95% HPD intervals) from bpp. Parameters for tree ii were estimated from all loci in chromosome 2L excluding 2La region, while those for tree xi were estimated from all loci in the Xag region of chromosome X. Divergence times were calculated assuming the mutation rate $2.8\times 10^{-9}$ per site per generation for autosomal noncoding loci (*A*), with 11 generations per year, and 0.524 and 0.323 times (supplementary fig. S4, Supplementary Material online) as large for the coding autosomes (*C*) and coding Xag loci (*D*), respectively. Ma, million years ago.

Systematically different species trees are inferred from different genomic regions for both data without the outgroup (fig. 1; supplementary table S2, Supplementary Material online) and those with the outgroup (supplementary fig. S1, Supplementary Material online). As in Fontaine et al. (2015), we recognize four regions of the genome with distinct phylogenetic relationships: 1) the majority of the autosomes and the pericentromeric region of the X chromosome, 2) the 2La inversion region, 3) the 3La inversion region, and 4) the Xag inversion region. In most parts of the autosomal genome (2L1, 2L2, 2R, 3L1, 3L2, and 3R), the maximum posterior probability (MAP) species tree is tree ii: (R(L(Q(A(GC))))), and less commonly, tree iii: (L(R(Q(A(GC))))) (fig. 1). The results are highly consistent between the noncoding and coding data. Tree ii is the autosomal tree obtained in recent studies using coalescent-based methods (Wen, Yu, Hahn, et al. 2016; Wen, Yu, and Nakhleh 2016). In contrast, the most common ML tree on the autosomes in the sliding-window analysis of Fontaine et al. (2015) is tree i, with the (RL) clade, which has near-zero posterior for almost all blocks (fig. 1). Note that species trees i, ii, and iii are three phylogenetic resolutions for R, L, and the clade (Q(A(GC))) around a very short branch at the root (fig. 2*A* and *C*). We demonstrate later that tree i, which is more balanced than tree ii, may be an artifact of the sliding-window approach used in Fontaine et al. (2015).

For the Xag region, the MAP tree from bpp for the noncoding data is predominantly tree xi: (R((L(AQ))(GC))). For the coding data, it is mostly tree x: ((L(AQ))(R(GC))) while tree xi is the MAP tree for two blocks (fig. 1; supplementary table S2, Supplementary Material online). In contrast, previous studies (Fontaine et al. 2015; Wen, Yu, Hahn, et al. 2016; Wen, Yu, and Nakhleh 2016) inferred tree ix: ((R(L(AQ)))(GC)) for the Xag region, with (GC) branching first. This tree is rarely supported in the bpp analysis of either noncoding or coding data sets, and we argue below that it is due to a bias of the sliding-window analysis. Note that trees ix, x, and xi represent the three relationships among the species/clades R, (GC), and (L(AQ)) around a very short branch at the root (fig. 2*B* and *D*). The noncoding loci are more divergent and more informative about the species phylogeny, and less affected by natural selection than the coding loci. Later we review other lines of evidence supporting tree xi, instead of trees ix or x, as the true species tree.

The two major autosomal inversion regions, 2La and 3La, are dominated by slightly different trees from the rest of the autosomes. In the 2La region, tree xii: (R(L(Q(G(CA))))) is the MAP tree in almost all blocks, which is almost exclusive to this part of the genome. We discuss the phylogeny of the 2La region later. The 3La region is dominated by tree viii: (L((RQ)(A(GC)))), with the (A(GC)) clade as in most parts of the autosomes, and the (RQ) clade, which suggests introgression between R and Q.

### Concatenation Produces Different Phylogenies from Coalescent-Based Methods

Although the sliding-window analysis of Fontaine et al. (2015) inferred tree i for the autosomes and tree ix for the Xag, our bpp analysis inferred trees ii and xi, respectively. Both the data and the analytical methods differ between the two studies. Instead of a 50-kb contiguous block in each sliding window of Fontaine et al. (2015), we used 100 widely spaced genomic segments (loci) in each block. Also we separated the noncoding and coding loci and realigned the sequences at each locus. To identify the factors that account for the different inferred trees, we used RAxML to infer one ML tree for each block, with all 100 loci in the block concatenated into a single alignment (of about 20 kb). *Anopheles christyi* is used as the outgroup to root the tree. For the autosomal noncoding data, the most common concatenation/ML tree is tree i (with frequency 46% vs. 23% for tree ii) (supplementary table S2 and fig. S2, Supplementary Material online). This is consistent with Fontaine et al. (2015) and different from the bpp analysis. For the autosomal coding data, the most common ML tree is tree ii (with frequency 48%) although it has a tendency to infer tree i as well (frequency 19%) (supplementary table S2, Supplementary Material online). This is more consistent with the bpp analysis. Whether the sequences were realigned or not did not impact the results (supplementary fig. S2, Supplementary Material online).

For the noncoding data from the Xag region, the most common ML tree for the realigned data is tree x (with frequency 53% vs. 37% for tree xi) (supplementary table S2 and fig. S2*A* and *B*, Supplementary Material online). For the coding Xag data, the most common ML tree for the realigned data is tree xi (with frequency 71% vs. 21% for tree ix). When the original genome alignments were used, the most common ML trees are trees ix and x for the noncoding data, and tree xi for the coding data (supplementary fig. S2*C* and *D*, Supplementary Material online). The sliding-window analysis of the noncoding data in the Xag region (Fontaine et al. 2015), which should be most similar to our concatenation analysis of the original alignments, indeed favored tree ix. Those results suggest that the most important factor accounting for the different trees between the two studies is the method used: The bpp coalescent method on one hand and the sliding-window/concatenation on the other. Both sliding-window and concatenation ignore the genealogical heterogeneity across the genome and fit a single tree to all sites in the alignment. Furthermore, alignment errors appear to have affected the ML analysis of the Xag data in Fontaine et al. (2015).

### Simulation Suggests Systematic Errors in Concatenation/Sliding-Window Analysis

To understand the differences between the MSC approach using bpp and concatenation using RAxML, we analyzed two sets of data simulated under the MSC model (Rannala and Yang 2003) and GTR + $\Gamma_{4}$ (Yang 1994a, 1994b). The first set is generated using tree ii, the autosomal bpp tree, with parameters estimated from the noncoding loci on 2L (2L1 + 2L2) under the MSC (the A00 analysis in Yang 2015) (fig. 2*A*). The second set is generated using tree xi, the Xag bpp tree, with parameters estimated from the Xag noncoding loci (fig. 2*B*). For each set, the same number of loci are simulated as in the real data, and analyzed in blocks of 100 loci in the same way as the real data.

The MAP species tree from bpp matches the correct tree ii in about 99% of the replicate data blocks for the 2L data, and about 85% for the Xag data (table 1). The posterior probability for the MAP tree is high when the tree is correct (median 0.99 for 2La and 0.84 for Xag) and is low when the MAP tree is wrong (median 0.65 for 2L and 0.67 for Xag). Even though bpp assumes the simple JC model (Jukes and Cantor 1969) while the data are simulated under the far more complex GTR + $\Gamma_{4}$ model, bpp performed well. Also 100 loci from the 2L region appear to be enough for bpp to infer the species tree with high confidence and high accuracy, but not for the Xag region, apparently because the Xag tree has an extremely short branch (fig. 2*B*).

**Table 1.** Proportions of Inferred Trees From Data Sets of 100 Loci Simulated Using Trees ii and xi (With the Minimum, Median and Maximum Support Values for the Inferred Tree in Parentheses).

Tree	bpp	RAxML (Subset 1)	RAxML (Subset 2)
2L data (6464 loci, 10 replicates)			
i	0.0062 (0.43, 0.65, 1.00)	0.4308 (0.34, 0.76, 1.00)	0.4492 (0.33, 0.76, 1.00)
ii*	0.9877 (0.47, 0.99, 1.00)	0.5139 (0.29, 0.77, 1.00)	0.5062 (0.31, 0.78, 1.00)
iii	0.0062 (0.48, 0.53, 0.81)	0.0385 (0.51, 0.56, 0.59)	0.0354 (0.36, 0.59, 0.94)
Xag data (1825 loci, 10 replicates)			
ix	0.1000 (0.42, 0.52, 0.78)	0.1105 (0.36, 0.61, 0.99)	0.1316 (0.38, 0.57, 0.96)
x	0.0474 (0.41, 0.67, 1.00)	0.4790 (0.46, 0.83, 1.00)	0.4632 (0.36, 0.83, 1.00)
xi*	0.8526 (0.38, 0.84, 1.00)	0.4105 (0.33, 0.75, 1.00)	0.4053 (0.38, 0.74, 1.00)

Note.—Each data set is a block of 100 loci. For bpp the inferred tree is the MAP tree and the support value is the posterior probability, while for RAxML the inferred tree is the ML tree from the concatenated alignments and the support value is the minimum bootstrap support value for clades. RAxML also inferred other trees in a small fraction (about 1%) of 2L data sets. Trees are given in figure 1. The correct tree (indicated by *) is tree ii for 2L data and tree xi for Xag data (fig. 1).

Concatenation/ML performed far more poorly than bpp. For the 2L data, the ML tree was the true tree ii about 51% of the time and the incorrect tree i about 44% of the time (table 1). Note that tree i has a more balanced shape, and concatenation is known to favor the incorrect, more balanced, tree when the true species tree is unbalanced with very short internal branches (Yang 2014, pp. 333–335). Tree i is the most common ML tree for the autosomes in the sliding-window analysis of Fontaine et al. (2015) and in our concatenation analysis of the noncoding data (supplementary fig. S2*A* and *C* and table S2, Supplementary Material online). For the Xag data, the ML tree was the correct tree xi about 41% of the time, and the incorrect tree x about 47% of the time (table 1). Again tree x is the most common ML tree in the analysis of the real Xag noncoding data (supplementary table S2 and fig. S2A and C, Supplementary Material online). Bootstrap support values for the ML trees are mostly moderate, probably because the phylogenetic signals from different loci appear conflicting to the method which attempts to fit one tree to all loci. Furthermore, the bootstrap values do not appear to depend on whether the ML tree is correct or wrong (table 1). The simulation results closely mimic the analysis of the real data and provide strong evidence that concatenation or sliding-window is unreliable for inferring the species tree in the *A. gambiae* species complex, and that tree i for the autosomes and tree ix for the Xag inferred in Fontaine et al. (2015) are artifactual.

### The X Chromosome Represents the True Species Phylogeny, With *A. merus* Diverging First

Fontaine et al. (2015) observed that the X chromosome (or more precisely the Xag region) and the autosomes support drastically different species trees and argued that the majority tree for Xag represents the true species branching order while the autosome trees are the result of introgression between species. Our analysis supports this assertion. This is consistent with the long-standing view that differentially fixed inversions on the X chromosome act as a reproductive barrier between species while the autosomes may be semipermeable and easily mixed among the three species *A. arabiensis*, *A. gambiae*, and *A. coluzzii* (Besansky et al. 2003; Wang-Sattler et al. 2007; Neafsey et al. 2010; O’Loughlin et al. 2014; Crawford et al. 2015). Nevertheless, our bpp analysis inferred different trees for both the autosomes and the Xag from those of Fontaine et al. (2015). Here, we summarize the evidence supporting the Xag tree as the true species tree, instead of the autosomal trees. We discuss further evidence (in addition to simulation discussed above) that the bpp tree for Xag (tree xi) is the true species tree, and that tree ix for the Xag and tree i for the autosomes inferred in Fontaine et al. (2015) are artifacts of the sliding-window analysis.

There are two major pieces of evidence in support of the Xag trees (e.g., tree xi) as the true species tree, rather than the autosomal trees (tree ii from bpp or tree i from Fontaine et al. 2015). First, the Xag trees are compatible with evidence on cross-species introgression. Note that the major difference between the Xag and autosomal trees concerns the relationships of *A. arabiensis* and *A. gambiae* + *A. coluzzii*. Introgression of *A. arabiensis* into the common ancestor of *A. gambiae* + *A. coluzzii* in any of trees ix, x, and xi (the three alternative trees for the Xag region) yields tree ii, the most common bpp tree for the autosomes. Introgression between *A. arabiensis* and *A. gambiae* + *A. coluzzii* has long been suggested (Besansky et al. 1994; García et al. 1996); this will be analyzed below through its impact on divergence times and through direct estimation of migration rates. In contrast, while tree x for the Xag could be explained by introgression of *A. merus* into the common ancestor of *A. gambiae* + *A. coluzzii* in tree ii if tree ii were the true species tree, evidence for such introgression is lacking.

Second, chromosomal inversions support the Xag trees and contradict the autosomal trees i and ii (Kamali et al. 2012; Fontaine et al. 2015). Ten fixed inversions have been identified in the *A. gambiae* complex, of which five are on the X chromosome. Comparison with the outgroup species revealed that the chromosomal orientations of *A. gambiae* and *A. merus* closely resemble the ancestral karyotype (Kamali et al. 2012; Fontaine et al. 2015), suggesting early divergences of *A. merus* and *A. gambiae* + *A. coluzzi*, as in the Xag trees. In contrast, *A. arabiensis* and *A. gambiae* + *A. coluzzii* differ by at least five overlapping inversions, with an intermediate orientation (X+) that is found in *A. melas* and *A. quadriannulatus* (Coluzzi et al*.* 1979, 2002; Fontaine et al. 2015). Moreover, *A. gambiae*+*A. coluzzii* and *A. merus* share the ancestral Xag orientation, and explaining the data using tree ii would require a reversal from the derived orientation X+ to the ancestral Xag in the lineage leading to *A. gambiae*+*A. coluzzii*. It is thus highly unlikely that *A. arabiensis* and *A. gambiae*+*A. coluzzii* are closely related, as in the autosomal trees.

Consideration of the statistical properties of the analytical methods suggests that tree ix for the Xag and tree i for the autosomes inferred in Fontaine et al. (2015) are artifactual. Our simulation has highlighted the systematic bias of the concatenated/ML method, which behaves similarly to the sliding-window approach of Fontaine et al. (2015): When the true tree is ii or xi, concatenation/ML tends to infer trees i and ix (or x), respectively (table 1). Consistent with this, we note that the Neighbor-Joining method applied to the average sequence divergences for the Xag region inferred tree xi, even though ML inferred tree ix (Fontaine et al. 2015, fig. 1*D*). Although ML applied to concatenated data may be inconsistent, average coalescent times or sequence divergences track species divergences, so that Neighbor-Joining (or UPGMA in the case where the molecular clock holds) is a coalescent-aware and statistically consistent method (Liu and Edwards 2009). Here we summarize further evidence against tree ix for the Xag and tree i for the autosomes.

First, tree i for the autosomes cannot be explained by introgression from *A. arabiensis* to *A. gambiae* + *A. coluzzii* alone. Indeed such introgression in any of the three alternative trees for the Xag (ix, x, and xi) leads to tree ii, the bpp tree for the autosomes.

Second, while chromosomal inversions favor the Xag trees over the autosomal trees, as discussed above, they support tree xi (the bpp Xag tree) far more strongly than tree ix (fig. 8*C* in Kamali et al. 2012). Indeed the most parsimonious tree for the fixed inversion data (Fontaine et al. 2015, fig. S27A) is (((QA)G)R), which is consistent with tree xi and requires no independent fixations of inversions in different lineages. In contrast, the inversion phylogeny that is consistent with tree ix (((QA)R)G), is not parsimonious and requires independent fixations in two lineages and introgression of 2La from *A. gambiae* to *A. arabiensis*, as well as ancient polymorphisms of the 2La and 2Ro inversions that likely predate speciation in the species complex (Fontaine et al. 2015, fig. S27B). Note that *A. merus* is the only species in the complex that has an ancestral 2Ro inversion and a derived 2Rp inversion on chromosome 2R, while the other species in the complex are fixed for the derived 2$R{+}^{o}$ and ancestral 2$R{+}^{p}$ orientations (Kamali et al. 2012). As a result, tree xi requires only one fixation event for each of those two inversions, whereas the other two trees (ix and x) require two independent fixations of 2$R{+}^{o}$ in two lineages, one leading to (GC) and another leading to (L(AQ)). We will discuss the phylogeny for the 2La region below. Our suggested species tree, tree xi, compared with tree ix inferred in Fontaine et al. (2015), provides a much simpler interpretation of the chromosomal inversion data.

### Divergence Times and Migration Rates Suggest A-to-G Introgression in Autosomes and R-to-Q Introgression in Chromosome 3L

**Fig. 3.**
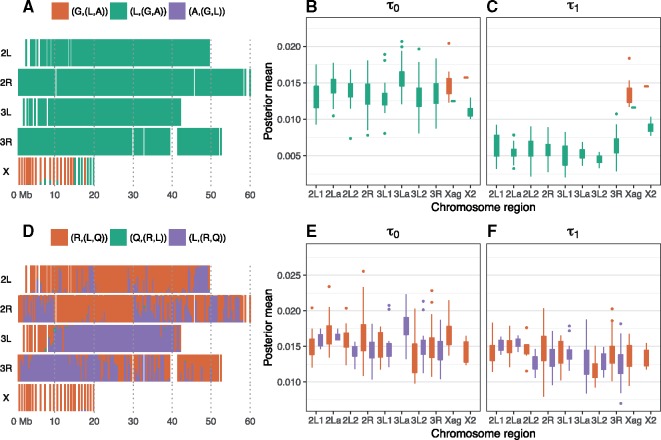
Bpp analysis of the GAL and RQL triplets. Left panel: posterior probabilities of species trees. Middle and right panels: posterior means of the two divergence times in the MAP species tree across different regions of the genome.

Our analysis of introgression has two components. First, following Fontaine et al. (2015), we estimate species divergence times as introgression has the effect of reducing divergence times between species (fig. 3). Second we apply the program 3s to data of species triplets to explicitly estimate the migration rates under the MSC model with migration (supplementary table S3, Supplementary Material online).

The autosomes and X chromosome support different trees for *A. gambiae*, *A. arabiensis*, and *A. melas* (fig. 1). In the bpp analysis of the GAL triplet data, the most common MAP species tree is (L(GA)) for the autosomes and (G(LA)) for Xag (fig. 3*A*), consistent with the bpp analysis of the full data of six species (fig. 1; supplementary table S2, Supplementary Material online). Fontaine et al. (2015) fitted ML trees to 10-kb nonoverlapping windows across the autosomes, and found that the divergence times in the tree (G(LA)) were greater than those in (L(GA)), suggesting that the reduced divergence is a consequence of autosomal introgression, and that the Xag tree represents the true species relationship, but the direction of the introgression was inconclusive (Fontaine et al. 2015, fig. 3). The same pattern is observed here. In the Xag tree (G(LA)), the two node ages are nearly identical, with $\tau_{0}\approx \tau_{1}$, while in the autosomal tree (L(GA)), the root age *τ*_0_ is close to the root age in the Xag tree, but *τ*_1_ is much smaller (fig. 3*B* and *C*). This is so even if we take into consideration the mutation rate variation among genomic regions (supplementary table S4, Supplementary Material online). This provides strong evidence that the Xag region is not affected by introgression and represents the true species relationship, while there is recent migration between *A. arabiensis* and *A. gambiae*+*A. coluzzii* for the autosomes (supplementary fig. S3, Supplementary Material online).

The A → GC introgression should lead to reduction of both *τ*_0_ and *τ*_1_ while the GC → A introgression should reduce *τ*_1_ only (supplementary fig. S3, Supplementary Material online). However, there may be little power to use this prediction to infer the direction of introgression, because 1) the gene trees are a mixture generated from the original species tree as well as the introgressed species tree; 2) the original species tree is star-like (fig. 2) with $\tau_{0}\approx \tau_{1}$ (supplementary fig. S3, Supplementary Material online) so that the two hypotheses make nearly identical predictions; and 3) the mutation rate varies among genomic regions (supplementary table S4, Supplementary Material online), complicating the comparison of *τ* estimates. Similarly the sliding-window analysis and the *D* statistic used by Fontaine et al. (2015) are uninformative about the direction of migration.

We use the 3s program to analyze the GAO, GAR, and GAL triplets, using *A. christyi* (O), *A. merus* (R), and *A. melas* (L) as the outgroup, respectively, to estimate explicitly the migration rates between the two ingroup species. For the GAO triplet, the estimates suggest G → A gene flow, but the evidence is not significant except for 2L and 3L (supplementary table S3, Supplementary Material online). No gene flow is detected in the opposite direction, nor for the X chromosome. However, since *A. christyi* is a very distant outgroup, our data in effect consist of species pairs and may not be informative (Dalquen et al. 2017). We thus analyzed the GAL and GAR triplets (supplementary table S3, Supplementary Material online). While *A. melas* is not a correct outgroup, both the correct species tree (G(LA)) and the incorrect tree (L(GA)) are close to the star tree (fig. 2), so that estimates from the wrong tree (L(GA)) may still be informative. Indeed the results for the GAL and GAR triplets are highly similar, suggesting strong evidence of introgression from *A. arabiensis* to *A. gambiae* affecting the autosomes but not the X chromosome. There is no evidence for migration from *A. gambiae* to *A. arabiensis*. The estimates from the GAR triplet may be the most reliable. Migration rate estimates vary considerably among chromosomal regions, which may reflect different strengths of natural selection removing immigrants, besides random sampling errors. The average rate for the autosomes from *A. arabiensis* to *A. gambiae* is *Nm* = 0.22 immigrants per generation (supplementary table S3, Supplementary Material online). For any plausible value of *N*, the migration proportion *m* must be orders of magnitude smaller than the recorded frequencies of hybridization (which is <0.1% but perhaps not much lower) (Coluzzi et al. 2002). The migration rate *Nm* represents the expected number of “successful” migrants, which are those that have contributed DNA in the recipient population after natural selection has removed unfit introgressed alleles or chromosomes.

With migration, estimates of *τ*_1_ in model M2 (gene flow) are greater than those under M0 (no gene flow), because ignoring migration leads to underestimation of the species divergence time *τ*_1_, as seen in the bpp analysis above (fig. 3). Strong positive correlation between the migration rate *M* and *τ*_1_ are thus expected. The estimates of *τ*_0_ (the age of the *A. gambiae* complex) are very similar between the two models and are also consistent with the estimates from bpp, in the range 0.012–0.014.

We analyzed the RQL triplet since there is evidence for R–Q introgression in chromosome arm 3L (fig. 1). Consistently with fig. 1, the MAP species tree is predominantly (R(LQ)) throughout the genome and in particular in the Xag region, but is (L(RQ)) in parts of 3L and 3R (fig. 3*D*). On average (R(LQ)) has older divergence times than the other two trees in most regions on the genome, except for chromosome 3L and the distal end of chromosome 3R (fig. 3*E* and *F*).

The likelihood ratio test applied to the RQO triplet data detected evidence for gene flow from R to Q in the autosomes, and in particular, in 3L, but the evidence is not significant (supplementary table S3, Supplementary Material online). The estimates of *τ*_0_ range over 0.0738–0.0925, comparable to those from the GAO triplet. Estimates of *τ*_1_ were 0.0102–0.0138, consistent with the bpp estimates on tree xi (0.0156 for Xag and 0.0138 for 2L) (fig. 2). As the LRT suffers from a lack of power due to the use of the distant outgroup, we analyzed the triplet RQL, treating L as the outgroup. Given that the species tree is star-like (fig. 2), we expect the estimates to be similar to those if the correct species tree were used. This analysis also suggests a small amount of gene flow from *A. merus* to *A. quadriannulatus* affecting the 3L region (supplementary table S3, Supplementary Material online).

### The Evolutionary History of the 2La Inversion Region

The 2La inversion is a *trans*-species paracentric chromosomal inversion in anopheles mosquitoes. It is polymorphic in *A. gambiae* and *A. coluzzii*, fixed for the ancestral 2La orientation in *A. arabiensis* and *A. merus*, and fixed for the derived $2L{+}^{a}$ orientation in *A. quadriannulatus* and *A. melas* (Coluzzi et al. 2002; Sharakhov et al. 2006). The 2La inversion is associated with malaria vectorial efficiency, adaptation to ecological habitats (in particular aridity) (Coulibaly et al. 2016; Ayala et al. 2017), and susceptibility to *Plasmodium* infection (Riehle et al. 2017). Sequence divergences in the 2La region are known to be greater between the karyotypes in *A. gambiae* and *A. coluzzii* (2La/2La, $2\text{La}/2L{+}^{a}$, $2L{+}^{a}/\;2L{+}^{a}$) than between species (Neafsey et al. 2010; O’Loughlin et al*.* 2014; Weetman et al. 2014; Fontaine et al. 2015; Riehle et al. 2017).

**Fig. 4.**
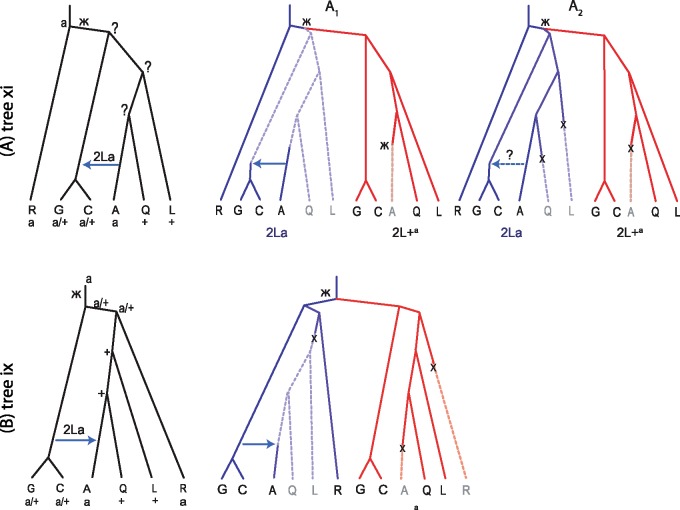
Species trees A (*top*) and B (*bottom*) for the 2La region (fig. S27*A* and *B* in Fontaine et al. 2015), based on the assumed species trees xi and ix, respectively. The inversion orientations in the extant and ancestral species are given as “a”: fixed for the 2La orientation, “+”: fixed for the 2L+^a^ orientation, and “a/+”: polymorphic for both orientations.

Species tree ix implies an evolutionary history for the 2La region, which is referred to here as tree B (fig. 4*B*). This was suggested by Fontaine et al. (2015, figs. 5*A* and S27*B*) and posits the existence of ancestral polymorphism of the two orientations prior to the radiation of the species complex, two independent losses of the $2L{+}^{a}$ orientation (in *A. merus* and *A. gambiae*), one loss of 2La, as well as GC → A introgression of the 2La orientation and complete replacement of the $2L{+}^{a}$ orientation in *A. arabiensis*. Our likelihood ratio test detected no such introgression, even though the same data provided overwhelming evidence for introgression in the opposite A → GC direction (supplementary table S3, Supplementary Material online). Similarly, a model of G → A introgression was found to be incompatible with the data in a simulation-based analysis of site-frequency spectrum data (He and Knowles 2016). Note that crossing experiments found evidence of introgression of 2La region from *A. arabiensis* into *A. gambiae* but not in the opposite direction (della Torre et al. 1997; Slotman et al. 2005).

**Fig. 5.**
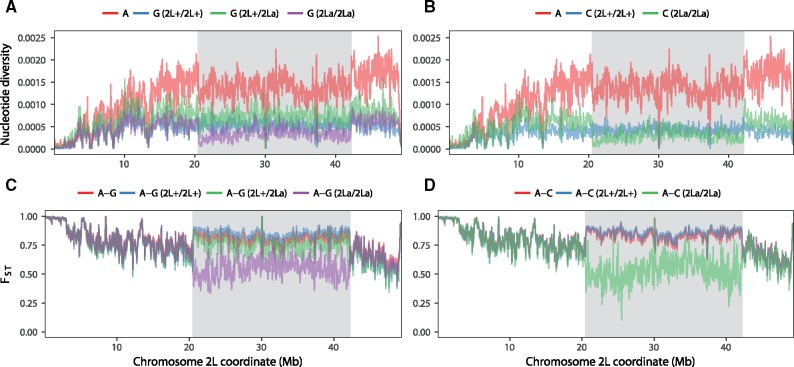
(*A* and *B*) Nucleotide diversity and (*C* and *D*) pairwise *F*_ST_ statistic between *A. arabiensis* (A) and different 2La karyotypes of *A. gambiae* (G) and *A. coluzzii* (C) calculated from genome-wide SNP data of natural populations from Fontaine et al. (2015). The 2La region is shaded. Sample sizes are *n *=* *23 for *A. gambiae* (35% $2L{+}^{a}$/$2L{+}^{a}$, 22% $2L{+}^{a}$/2La, 43% 2La/2La), *n *=* *11 for *A. coluzzii* (73% $2L{+}^{a}$/$2L{+}^{a}$, 27% 2La/2La, no $2L{+}^{a}$/2La) and *n *=* *12 for *A. arabiensis*.

Our proposed species tree (tree xi) suggests an alternative history for the 2La region, tree A (fig. 4*A*), which is more parsimonious (Fontaine et al. 2015, fig. S27*A*). This posits the origin of the derived $2L{+}^{a}$ form after *A. merus* branched off, and A → GC introgression. This introgression may predict less polymorphic 2La orientation than $2L{+}^{a}$ in *A. gambiae* and *A. coluzzii*. Indeed a reduced nucleotide diversity in the 2La region in the 2La/2La karyotype of *A. gambiae* and *A. coluzzii* is observed but not in the $2\text{La}/2L{+}^{a}$ and $2L{+}^{a}/\;2L{+}^{a}$ karyotypes (fig. 5*A* and *B*). Such differences in nucleotide diversity among different karyotypes may not be predicted by tree B since all three karyotypes in *A. gambiae* and *A. coluzzii* are old under tree B. Also there is no clear reduction in nucleotide diversity in the 2La region in *A. arabiensis*, as may be predicted by tree B. Genetic differentiation measured by *F*_ST_ is reduced between *A. arabiensis* and the 2La/2La karyotype of *A. gambiae* and *A. coluzzii*, but not for the other pairs, relative to the rest of the chromosome (fig. 5*C* and *D*), although this pattern is predicted by both trees as a consequence of 2La introgression. Moreover, the frequency of the 2La orientation in *A. gambiae* is higher in geographical ranges where *A. gambiae* overlaps with *A. arabiensis* (He and Knowles 2016), consistent with tree A, but not expected under tree B. As mentioned above, evidence for the A → GC introgression is well-known (della Torre et al. 1997; Slotman et al. 2005).

We consider two variations of tree A: A1 and A2. Tree A1 requires one inversion of 2La into $2L{+}^{a}$ after *A. merus* branched off and one reversal of $2L{+}^{a}$ to 2La in the lineage leading to *A. arabiensis*, with the polymorphism of 2La region in *A. gambiae* + *A. coluzzii* explained by the A → GC introgression. Since the reversal to 2La occurred in a homogeneous background in *A. arabiensis*, the breakpoints of 2La in *A. arabiensis* are expected to differ from those in *A. gambiae* + *A. coluzzii* and *A. merus*. However, the organization of genes and noncoding elements around the 2La breakpoints in those species appear to be identical (Sharakhov et al. 2006), suggesting that multiple origins of the 2La orientation in this species complex are very unlikely.

Tree A2 assumes an extensive period of ancestral polymorphism of both orientations, and independent losses of the 2La orientation in *A. melas* and *A. quadriannulatus*, and a loss of the $2L{+}^{a}$ orientation in *A. arabiensis*. This allows A → GC introgression of the 2La orientation but does not require it. The introgression in a polymorphic background should result in two distinct haplotypes of the 2La orientation in *A. gambiae* and *A. coluzzii* (the original and introgressed). However, no such heterogeneity has been found in the 2La heterozygotes in analysis of genome-wide SNP data from hundreds of field-caught mosquitoes of *A. gambiae* and *A. coluzzii*, apart from clustering by geographical origins (Riehle et al. 2017; The *Anopheles gambiae* 1000 Genomes Consortium 2017), which supports a scenario of no A → GC introgression of 2La. A variation to tree A2 is to allow an additional loss of the 2La orientation in the *A. gambiae* + *A. coluzzii* ancestor followed by introgression of 2La from *A. arabiensis*. Those hypotheses make different predictions about sequence divergences between species and between the different karyotypes of *A. gambiae* and *A. coluzzii*, which may be useful to distinguish between them. The data of Fontaine et al. (2015), in the form of haploid consensus sequences generated from the diploid samples, may not have such resolution.

### Estimation of Species Divergence Parameters

Even though the data we used are a small percentage of the whole genome, parameter estimates are typically very precise, with small 95% highest posterior density (HPD) intervals. Exceptions are the population size parameters $\theta_{G}$ and $\theta_{C}$ for *A. gambiae* and *A. coluzzii*, which are a few folds larger than for other populations. Nonetheless, the estimates are comparable with recent estimates based on genome-wide SNP data from diverse population samples (The *Anopheles gambiae* 1000 Genomes Consortium 2017).

The bpp estimates of *τ* are proportional between the coding and noncoding data, with the regression coefficient 0.524 (with $r^{2}=0.998$) for tree ii for the 2L region and 0.323 ($r^{2}=0.994$) for tree xi for the Xag (supplementary fig. S4*A* and *B*, Supplementary Material online). The role of purifying selection removing nonsynonymous mutations is predominantly the reduction of neutral mutation rate in the coding regions, highlighting the utility of the coding loci (just like the noncoding loci) in MSC-based analysis (Shi and Yang 2018).

The estimates of *τ*s are also largely proportional between tree ii for the 2L and tree xi for the Xag (after the GC clade is removed to make the two trees identical). The slope is 1.060 for the noncoding loci ($r^{2}=0.989$ using four pairs of *τ*) (supplementary fig. S4*C*, Supplementary Material online), suggesting that the X chromosome have a slightly higher mutation rate than those on the autosomes (supplementary table S4, Supplementary Material online). For the coding loci, the *τ* estimates are also nearly proportional but the slope is 0.674 (with $r^{2}=0.997$) (supplementary fig. S4*D*, Supplementary Material online), indicating that the coding regions in the Xag region are more conserved than those in the autosomes.

**Fig. 6.**
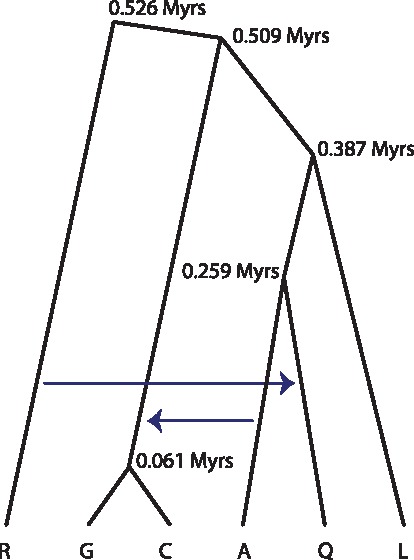
Estimated species phylogeny with introgression for the *A. gambiae* species complex. Branch lengths are based on the divergence time estimates (*τ*s) from the Xag data (fig. 2*B*).

To translate the estimates of *τ*s and *θ*s into geological times and population sizes, a mutation rate has to be assumed. As no mutation rate estimates are available for the *Anopheles*, we use the *Drosophila* rate of $2.8\times 10^{-9}$ mutations per site per generation (Keightley et al. 2014), with 11 generations per year. This places the root of the *A. gambiae* species complex at 0.526 My, the divergence of the GC clade from the (L(AQ)) clade at 0.509 My, and the divergence of *A. gambiae* and *A. coluzzii* at 0.061 My (fig. 6). The G–C divergence time is expected to be a serious underestimate, because our analysis has ignored the introgression between *A. gambiae* and *A. coluzzii*, which should cause those sister species to be preferentially grouped together (Leaché et al. 2014) and which should cause underestimates of their divergence time. If we use the mutation rate of $5.5\times 10^{-9}$ instead (Schrider et al. 2013), the ages will be younger by a half. Our age estimates are much younger than those in Fontaine et al. (2015), which estimated the age of the clade to be 1.85 My. Using a mutation rate of $1.1\times 10^{-9}$ with 10 generations per year as in Fontaine et al. (2015) will give 1.47 My for the age of the clade, still 20% younger. The concatenation/ML analysis misinterprets sequence divergence as species divergence and may be expected to overestimate node ages. Furthermore, sliding-window or concatenation analysis produces systematically biased estimates of species divergence times and population sizes because it incorrectly attributes the genealogical heterogeneity across the genome as variation in evolutionary rate among sites (Ogilvie et al. 2017).

It has been argued that increased anthropophily could not have evolved in *A. gambiae* and *A. arabiensis* before humans evolved to high densities after the advent of agriculture and the clearing of the forest in Africa, and that the ability to effectively transmit human malaria must be a relatively recent trait in the complex, less than 5000 years ago (Coluzzi et al. 2002; Ayala and Coluzzi 2005). It is not yet clear which event on the species phylogeny coincides with the acquisition of malaria vectorial capacity. If this is prior to the divergence of *A. gambiae* and *A. coluzzii*, both of which are highly anthropophilic, the mutation rate would need to be at least two orders of magnitude higher to bring our age estimates in line with such recent dates.

## Discussion

### Introgression in the *A. gambiae* Species Complex

Inference of migration or introgression among species in the *A. gambiae* species complex is important both for a reliable estimation of the species phylogeny and for understanding the mode and rate of migration itself. In this study, we have used the ML program 3s to test for gene flow using multiloci sequence data. There are a number of differences in our results compared with tests of introgression conducted by Fontaine et al. (2015) using the *D* statistic (Green et al. 2010; Durand et al. 2011) and by Wen, Yu, Hahn, et al. (2016) using the phylonet program. Here we discuss the methodological differences among those inference methods before their differences in results when they are applied to the mosquitoes data.

The 3s program (Zhu and Yang 2012; Dalquen et al. 2017) is an ML implementation of the IM model (Hey and Nielsen 2004). It is limited to three species (*S*_1_, *S*_2_, and *S*_3_), with the fixed phylogeny ((*S*_1_, *S*_2_), *S*_3_). Migration is allowed between species *S*_1_ and *S*_2_, whereas outgroup species *S*_3_ is used to improve the power of the test (Zhu and Yang 2012; Dalquen et al. 2017). It is designed to analyze multiloci sequence data, with only three or two sequences per locus. It allows asymmetrical migration rates: *M*_12_ and *M*_21_, where $M_{ij}=N_{j}m_{ij}$ is the expected number of migrants from species *i* to *j* per generation, with *m_ij_* to be the proportion of immigrants in species *j* from species *i*. The program can be used to infer the direction of migration and to estimate the migration rates. The distribution of the gene trees and coalescent times is given by the MSC model with migration, whereas the likelihood is calculated under the molecular clock and the JC mutation model. Averaging over the gene trees at each locus is achieved analytically, whereas the coalescent times are integrated out numerically using Gaussian quadrature. A likelihood ratio test is used to detect gene flow by comparison with a null model that assumes zero migration rates. Thus 3s is a full-likelihood implementation of the IM model. Although it is limited to three species, it can handle tens of thousands of loci. Note that the test can be conducted using species *S*_1_ and *S*_2_ only, but including a third species *S*_3_ improves the power of the analysis considerably (Dalquen et al. 2017).

The *D* or ABBA-BABA statistic (Green et al. 2010; Durand et al. 2011) is applied to data from four species, with the fixed phylogeny $\left(((S_{1},S_{2}),S_{3}),O\right)$. Here *O* is the outgroup while the focus is on the possibility that *S*_3_ may exchange migrants with *S*_1_ or *S*_2_. The test is based on the expectation that if there is ILS but no gene flow, the two parsimony-informative site patterns that are conflicting with the species tree (ABBA and BABA) should occur with equal frequency, and a significant difference between the two site-pattern counts (*n_ABBA_* and *n_BABA_*), or equivalently between $D=\frac{n_{ABBA}-n_{BABA}}{n_{ABBA}+n_{BABA}}$ and 0, will be evidence for gene flow. Thus *D* can be used to test for gene flow against the null expectation of no gene flow. Note that the null hypothesis (*D* = 0) is compatible with gene flow between *S*_1_ and *S*_2_, or between *S*_3_ and the ancestor of *S*_1_ and *S*_2_ (Durand et al. 2011). The *D* statistic is similar to 3s in that both test for gene flow between two species although *D* relies on the outgroup species *O* to infer the ancestral nucleotide, while 3s assumes the molecular clock and does not require an outgroup. A major consequence of gene flow impacting the neutral genome is that different regions of the genome will have different distributions of gene genealogies (gene tree topologies and coalescent times), depending on whether or not sequences from that genomic region are transferred across species. There should be rich information concerning gene flow in the genealogical heterogeneity across the genome. This information is fully used by 3s and completely ignored by the *D* statistic. Such information loss must be a major reason for the fact that the *D* statistic is uninformative about the direction of gene flow and cannot be used to estimate the migration rates.

Fontaine et al. (2015, fig. S25) applied the *D* statistic to test for gene flow using the data of genome-wide SNPs, which should be equivalent to the use of sequences since *D* depends only on counts of two site patterns: ABBA and BABA. This analysis inferred introgression between A and G + C (direction unknown) on chromosomes 2R, 3L, and 3R, but not on 2L. Using 3s we detected significant gene flow from A to G + C throughout the autosomes, particularly in the 2La region, but no gene flow from G + C to A. The *D* statistic also suggested gene flow between Q and R, particularly on 3L, and between Q and G + C on all chromosomes. There appears to be no evidence supporting gene flow between Q and G + C, and Fontaine et al. (2015) attributed the result to “side-effects of the massive introgression between A–C and A–G”. Based on simulations Zheng and Janke (2018) suggested the rule of thumb that the *D* statistic may not be reliable when the population size is larger than half the species divergence time in generations (or $\theta >2\tau_{0}$ in the notation of this study). According to this the *A. gambiae* species complex is at the borderline of reliability for the *D* statistic (see fig. 2).

The phylonet program (Wen, Yu, Hahn, et al. 2016; Wen, Yu, and Nakhleh 2016) takes inferred gene tree topologies for the different loci as input data and fits models of introgression. This method can identify the direction of gene flow and estimate the strength of introgression as reflected in the inheritance probability parameter at each hybridization event. There are, however, two serious weaknesses with this method. First it ignores information in the branch lengths (or coalescent times) in the gene trees. Second, it fails to accommodate phylogenetic reconstruction errors in inferred gene trees. Such errors or uncertainties are substantial for closely related species with very high sequence similarities and can cause the heuristic summary methods to have much poorer statistical properties than full likelihood methods (Xu and Yang 2016).

The phylonet program applied to the genomic sequence data of the *A. gambiae* species complex inferred multiple introgression events. The analysis appeared to be sensitive to the assumed species phylogeny. Using the phylogeny of Fontaine et al. (2015), the program detected strong introgression with inheritance probability >90% between A and G + C (in both directions), and moderate levels of introgression with inheritance probability 20%–30% between R to Q (in either direction depending on the details of the analysis) for the autosomes (Wen, Yu, Hahn, et al. 2016, figs. 3 and 5). Note that we detected strong gene flow from A to G + C but no gene flow in the opposite direction. When the species phylogeny was not fixed, the program detected gene flow from Q to G, from Q to R, from A to G + C, and from L to R (Wen, Yu, Hahn, et al. 2016, fig. 6). Some of those inferences may be spurious: For example, L and R do not overlap geographically.

At any rate, our analysis of gene flow in the *A. gambiae* species complex in this study has been tentative, focused on its impact on the species phylogeny. The 3s program we used is limited to three species. Furthermore, we did not consider the variation in migration rate among geographical populations, and we did not distinguish between one-off introgression (hybridization) and continuous-time migration. A more detailed analysis is warranted when more high-quality data and powerful methods are available. We note that full likelihood methods for inferring introgression, taking sequence alignments as the data and thus making use of information in gene trees while accommodating their uncertainties, are under active development (Wen and Nakhleh 2018; Zhang et al. 2018). Those methods use MCMC to average over genealogical histories at each locus and are currently impractical for large data sets (with >100 loci, say) due to mixing problems, but future improvements may make them practical for genome-scale data sets.

### The *A. gambiae* Phylogeny Provides a Framework for Studying the Evolution of Ecological and Epidemiological Characters

Although we support the major conclusion of Fontaine et al. (2015) that the Xag region of the X chromosome represents the species branching order, and the conflicting autosome phylogenies are a result of extensive introgression, the inferred species trees are different: Fontaine et al. (2015) inferred tree ix for the Xag, while we inferred tree xi, with *A. merus* diverging first.

To examine the robustness of our bpp analysis, we conducted two further simulation experiments. The first examines whether bpp is biased toward trees ii and xi, with data simulated assuming species trees i and ix, as suggested by the concatenation analysis and by Fontaine et al. (2015). Parameters for species tree i were estimated using bpp (the A00 analysis) from the 2L loci, and for species tree ix from the Xag loci, similar to table 1. The GTR + $\Gamma_{4}$ model was used to generate the data, which was analyzed using bpp under JC and using concatenation/RAxML under GTR + $\Gamma_{4}$. The results are summarized in supplementary table S5, Supplementary Material online.

The internal branch in tree i leading to the (LR) clade estimated from the 2L data was $\tau_{0}-\tau_{RL}=0.000009$ (supplementary fig. S6, Supplementary Material online), so that the true species tree in the simulation had nearly a trichotomy. All three resolutions of the branch (trees i, ii, and iii) were supported by bpp in substantial proportions of the data sets (of 100 loci), while concatenation/RAxML favored the more balanced tree i. The fact that concatenation outperforms bpp in this case is reminiscent of the complexity of phylogenetic tree estimation discussed by Yang (1997). When a data set of 1,000 loci was analyzed using bpp, the true tree i dominated, with the posterior at ∼100%, suggesting that bpp is statistically consistent for this species tree despite the very short internal branch.

For the Xag data, the internal branch in species tree ix leading to the (RLAQ) clade was $\tau_{0}-\tau_{RLAQ}=0.000021$ (supplementary fig. S6, Supplementary Material online). Again bpp supported all three resolutions of that short branch (trees ix, x, and xi) in substantial proportions of the data sets, while concatenation/RAxML favored the more balanced tree x. However, when a replicate data set of 1,000 loci was analyzed using bpp, the resulting species tree was tree x, with posterior at ∼100%. It is unclear whether bpp is inconsistent for this tree because of model misspecifications (Yang and Zhu 2018). We conclude that bpp inferred species trees ii and xi from the genomic data not because it is biased toward those trees.

The second simulation is designed to examine whether the *A. arabiensis* to *A. gambiae* + *A. coluzzii* gene flow, as inferred from the genomic data using 3s, is sufficient to misled phylogenetic-reconstruction and species-tree methods to generate wrong trees if the true species tree is tree xi. We simulated data using tree xi for the Xag and the parameter estimates on tree xi, but with migration from *A. arabiensis* to the common ancestor of *A. gambiae* + *A. coluzzii* at the rate of 0.22 migrants per generation. We then analyzed the data using bpp and concatenation/ML. Both methods produced tree ii as the best estimate in every block of 100 loci. The results suggest that the level of introgression estimated from the autosomes is indeed sufficient to mislead phylogenetic and species-tree methods to infer an incorrect phylogeny, a scenario discussed by Mallet et al. (2016, figure 3).

As species tree xi is consistent with the chromosomal inversion phylogeny, it leads to a more parsimonious interpretation of the inversion data, removing the need to assume introgressions for which no evidence is available, such as the introgression from *A. gambiae* + *A. coluzzii* to *A. arabiensis* (Fontaine et al. 2015). Recently Anselmetti et al. (2018) used the autosomal and X chromosomal trees of Fontaine et al. (2015) as given and the RAxML gene trees for coding loci as input data in their genome assembly pipeline to improve scaffolding of contigs and to infer chromosomal rearrangement events from the resulting assemblies. The analysis appears to be sensitive to the assumed species tree (and errors in the inferred gene trees) and should be redone in light of the new species phylogeny.

The knowledge of the species phylogeny provides the necessary framework for studying the evolution of major ecological characters. Physiological adaptation to breeding in saltwater in *A. merus* and *A. melas* must have evolved independently, as postulated early (Coluzzi et al. 1979; Kamali et al. 2012). It was suggested that vectorial capacity has evolved multiple times, in *A. gambiae*, *A. merus*, *A. arabiensis*, and the lineage that led to *A. quadriannulatus* and *A. melas* (Kamali et al. 2012). This interpretation needs to be modified in light of the species phylogeny. It is possible for vectorial capacity to originate only once, in the common ancestor of the *A. gambiae* complex and then lost in *A. quadriannulatus*. We note that reconstruction of a single ecologically important character may involve high uncertainties. Identifying genes or amino acid changes that may be responsible for vectorial capacity and dating the speciation and introgression events (e.g., by obtaining more relevant mutation rate estimates) may provide valuable information.

### The Importance of Coalescent-Based Methods to Inferring Challenging Species Trees Resulting from Radiative Speciations

Although theoretical studies have suggested that concatenation may be unreliable and even inconsistent when the species tree contains short internal branches and large ancestral populations (Kubatko and Degnan 2007; Roch and Steel 2015), real data examples are relatively rare (Giarla and Esselstyn 2015; Shi and Yang 2018). The *A. gambiae* species complex appears to be such a case and serves to illustrate the importance of properly accounting for ILS in such analysis and the power of full-likelihood coalescent-based methods in resolving such difficult species phylogenies. Our analysis of both the real and simulated data suggests that the JC mutation model assumed in 3s and bpp is adequate for multiple-hit correction and recovers the true species tree even in data sets simulated under a complex GTR + $\Gamma_{4}$ model. Note that sequence divergence between the species in the complex is within 5% (supplementary fig. S5, Supplementary Material online). The molecular clock assumption also approximately holds as the species are closely related.

The impacts of various factors in the inference of shallow species trees, including sequence divergence, model assumptions, recombination, and noncoding versus coding data partitioning have been discussed in detail in Shi and Yang (2018, and references therein). Evidently accommodating the gene tree/species tree conflicts and introgression has the greatest impact on the analysis. Although the early phylogenomic study (Fontaine et al. 2015) emphasized ILS, the analytical methods used ignored it. We expect such methodological differences will be important in other similar challenging species tree problems. Analysis of large genome-scale data sets requires a proper statistical framework.

The data we analyzed, which consist of widely separated genomic segments from the genome, constitute a small fraction of the genome (21.6 Mb/278 Mb =7.8%). However, with so many loci, the species trees can be resolved with high confidence and accuracy. The sliding-window analysis (Fontaine et al. 2015) uses more data in terms of base pairs, but it ignores the gene-tree heterogeneity across the genome and may be misled by ILS.

The agreement of our inferred species tree, tree xi based on the genomic sequences from the Xag region of the X chromosome, with the chromosomal inversion phylogeny of Kamali et al. (2012) highlights the utility of both chromosomal rearrangements and genomic sequence data in resolving the challenging phylogeny of the *A. gambiae* complex. As pointed out before (White et al. 2011; Kamali et al. 2012), sequence data tend to have weak phylogenetic information when the species are closely related and the sequences are highly similar. However, the number of characters is huge. Chromosomal rearrangements represent rare or even unique events, which make each character highly informative. However, they may often be compatible with multiple interpretations. It has been common to assume that species sharing inversions form a clade or are sister taxa (White et al. 2011; Kamali et al. 2012), but such inference is not safe when the original and derived states of the inversion are unknown and the inferred tree is unrooted. For a long time *A. quadriannulatus* was considered the closest species to the ancestral lineage because it has a large number of hosts, feeds on animal blood, tolerates temperate climates, exhibits disjunctive distribution, and possesses a “standard” karyotype (Coluzzi et al. 1979, 2002). However, this was based on misinterpretations of the unrooted phylogeny. Our analysis highlights the importance of integrating the different sources of information (from sequences, chromosomal inversions, and introgression) to resolve this challenging species tree problem.

## Materials and Methods

### Data sets

We obtained the whole genome alignment from Fontaine et al. (2015) (doi: 10.5061/dryad.f4114) for six species in the *A. gambiae* species complex: *A. gambiae* (G), *A. coluzzii* (C), *A. arabiensis* (A), *A. melas* (L), *A. merus* (R), and *A. quadriannulatus* (Q), as well as the *A. gambiae* PEST reference genome and two Pyretophorus outgroup species (*A. christyi* and *A. epiroticus*). For each of the six ingroup species, there are two genomes, one from a laboratory colony (reference genome) and another from a field-collected individual (nonreference genome). We used twelve whole genomes for the six ingroup species, and *A. christyi* (O) genome as an outgroup, excluding *A. gambiae* PEST and *A. epiroticus* genomes from our analysis. There are thus 12 sequences per locus or 13 if the outgroup is included. The original alignment is partitioned into 2L, 2R, 3L, 3R, and X chromosomal arms. We further separate out three main inversion regions 2La, 3La, Xag (in chromosomes 2L, 3L, and X, respectively) using breakpoint coordinates from table S11 in Fontaine et al. (2015), resulting in ten chromosomal regions: 2L1, 2La (the inversion region on 2L with coordinates 20.5–42.1 Mb), 2L2, 2R, 3L1, 3La (the inversion region on 3L with coordinates 14.5–35.6 Mb), 3L2, 3R, Xag (the inversion region about 14.8 Mb on the distal end of the X chromosome), and X2 (the pericentromeric region of the X chromosome with coordinates 14.8–24 Mb). The distal end of the X chromosome contains a small region of about 21 kb outside of the Xag inversion, which may not be very informative about the species tree, and is thus combined into the Xag region here.

The MSC model implemented in bpp and 3s assumes free recombination among loci and no recombination within a locus. Thus the ideal loci for this kind of analysis are short genomic segments that are far apart so that recombination within a locus can be ignored while recombination between loci is so common that the different loci have nearly independent histories (Burgess and Yang 2008; Lohse et al. 2011).

We used the gene set annotation of *A. gambiae* PEST strain (AgamP3 assembly) from VectorBase to split the alignment into coding and noncoding regions. For the noncoding regions, we split the alignment for each chromosomal region into smaller segments (loci) using the ambiguous nucleotide character (*N*) as breakpoints. Each locus is between 100 and 1,000 bases and has fewer than 50% gaps, and two consecutive loci are at least 2 kb apart. In a preliminary analysis, we also compiled data with a minimum gap of 10 kb between loci and the results were very similar. Thus we used 2 kb to preserve more loci. There are 57,592 noncoding loci in total. Manual inspection suggests that a number of regions appear to be misaligned in the original whole genome alignment (Fontaine et al. 2015). Thus we realigned all loci using MAFFT (Katoh and Standley 2013), using the iterative refinement method (the L-INS-i option). This appears to fix the alignment errors. After removing gaps, each locus has between 11 and 973 sites (median 195). The number of parsimony-informative sites ranges from 0 to 229 (median 15). For the coding regions, we also require each locus, which is a part of an exon, to have length at least 100, and contain fewer than 50% gaps. But unlike the noncoding loci, we do not constrain the maximum length of each locus or the minimum distance between loci. There are 23,505 coding loci in total. Each locus ranges from 52 to 6,541 sites (median 210). The number of parsimony-informative sites ranges from 0 to 403 (median 6). All processing of the original genome alignment data was done using custom python scripts.

### Species Tree Estimation Using Bpp and Concatenation

We inferred the species tree among the six ingroup species using two methods: 1) the Bayesian MSC-based method implemented in bpp v.3.4 (Rannala and Yang 2003, 2017; Yang and Rannala 2014; Yang 2015) assuming the JC mutation model (Jukes and Cantor 1969) and 2) concatenation and ML under the GTR + $\Gamma_{4}$ model using RAxML v.8.2 (Stamatakis 2014). To reduce the computation cost, we partitioned the data into blocks of 100 loci in each chromosomal region, resulting in 582 blocks for the noncoding data, and 239 blocks for the coding data. Each block was analyzed separately, treated as 100 loci with independent genealogical histories by bpp and as one supersequence by RAxML.

The MSC model accommodates ancestral polymorphism and deep coalescence, and the likelihood implementation in bpp accounts for phylogenetic uncertainties at each locus. The parameters under the MSC include $\Theta =(\tau_{i},\theta_{i})$, where *τ_i_* is the species divergence time, *θ_i_* is the population size parameter. Inverse gamma priors were assigned on *τ* and *θ* parameters. For the noncoding data, we used θ∼InvG(3,0.04) for all populations, which has mean 0.02, and the root age $\tau_{0}∼\text{InvG}(3,0.2)$, which has mean 0.1. Given *τ*_0_, species divergence times for nonroot nodes are uniform on the interval $\left(0,\tau_{0}\right)$, generated from the symmetric Dirichlet distribution (Yang and Rannala 2010). These *θ* and *τ* parameters are in the units of the expected number of mutations per site. To convert these parameters to actual times (before present) and actual population sizes, we use the mutation rate estimates for *Drosophila*: $2.8\times 10^{-9}$ (Keightley et al. 2014) and $5.5\times 10^{-9}$ (Schrider et al. 2013) mutations per site per generation, with 11 generations per year (The *Anopheles gambiae* 1000 Genomes Consortium 2017). Thus the population sizes have prior mean of about 0.91 or 1.79 million individuals, and the root divergence time has prior mean of about 1.65 or 3.25 million years. For the coding data, we used $\theta_{i}∼\text{InvG}(3,0.008)$ and $\tau_{0}∼\text{InvG}(3,0.04)$, which have five times smaller means than for the noncoding data. The species tree prior was the uniform distribution over rooted trees (Yang and Rannala 2014). We initially estimated the species tree with *θ* integrated out analytically, as this improves the mixing property of the algorithm (A01 analysis in Yang 2015). We then estimated the population size parameters and species divergence times for each of the most likely species trees (A00 analysis in Yang 2015).

The MCMC was run for $2\times 10^{6}$ iterations after $4\times 10^{4}$ iterations of burn-in. Samples were recorded every 20 iterations. For each block of loci, two independent runs were performed using different starting trees. Convergence was assessed mostly by checking for consistency between runs in posterior probabilities for species trees. If the MAP trees from the two runs were the same, we required their posterior probabilities to differ by ≤0.3, while if the MAP trees were different, we required the mean absolute difference in the posterior probabilities to be ≤0.3. We then combined samples from the two runs to produce the posterior summary. Otherwise, we repeated the two runs until convergence was achieved.

For parameter estimation on a fixed species tree (A00 analysis), we also included the outgroup species (*A. christyi*) in the data since estimated parameters will be used later in simulation experiments. We performed ten independent runs of MCMC, each with 10^6^ iterations after a burn-in of $4\times 10^{4}$ iterations.

For the concatenation analysis, we merged each block of 100 loci into a single alignment and then ran RAxML. We also split each alignment into two subsets, containing only either reference genomes or genomes from natural population samples. We used the GTR + $\Gamma_{4}$ model and performed 100 independent runs with random starting trees (option –*N* 100) to infer the ML tree. The number of bootstrap replicates was 100.

### Generation and Analysis of Simulated Data Sets

Our Bayesian analyses suggest tree ii for the autosomes and tree xi for the Xag region of the X chromosome, while the sliding-window analysis of Fontaine et al. (2015) favored trees i and ix, respectively. To investigate those differences, we used the MCcoal program in bpp v.3.4 to simulate two data sets using trees ii and xi, each with ten replicates. We performed bpp and concatenation/RAxML analyses on the simulated data sets in the same way as for the real data sets.

For tree ii, we simulated 6,464 loci under the GTR + $\Gamma_{4}$ model, each of length 200. We used the posterior means of *τ*s and *θ*s in the MSC model obtained from the real 6,464 loci from chromosome 2L, exclusive of 2La region (fig. 2). The parameters of the GTR + $\Gamma_{4}$ model for “evolving” sequences given the gene tree were allowed to vary among loci. For each locus, the base frequencies π =$\left(\pi_{T},\pi_{C},\pi_{A},\pi_{G}\right)$ were generated from a Dirichlet distribution $\pi ∼\text{Dir}(\alpha_{T},\alpha_{C},\alpha_{A},\alpha_{G})$ with parameters $\left(\alpha_{T},\alpha_{C},\alpha_{A},\alpha_{G}\right)$ =(20.49, 21.22, 20.46, 20.97). These were the ML estimates obtained when the Dirichlet distribution was fitted to the observed base frequencies. The exchangeability parameters q=(a,b,c,d,e,f) for the GTR model (Yang 1994a) were also generated from a Dirichlet distribution $q∼\text{Dir}(\alpha_{a},\alpha_{b},\alpha_{c},\alpha_{d},\alpha_{e},\alpha_{f})$ with parameters $\left(\alpha_{a},\alpha_{b},\alpha_{c},\alpha_{d},\alpha_{e},\alpha_{f}\right)$ =(7.59, 3.23, 2.95, 2.93, 2.93, 7.57), estimated by fitting the Dirichlet distribution to the RAxML estimates of *q* for the data at each locus. The overall rate for each locus was generated from *G*(5, 5). The shape parameter *α* = 5 was based on fitting the gamma distribution G(α,β) to locus-wise estimates of the tree lengths from RAxML. The alignment of sequences at each locus is not always informative enough to estimate the *α* parameter of GTR + $\Gamma_{4}$ model. Instead, we generated *α* for each locus from *G*(20, 4), with mean 5.

Similarly for tree xi, we simulated 1,825 loci each of length 200, where the species-tree parameters (*θ*s and *τ*s) were estimated using the real 1,825 loci from the Xag region (fig. 2). We used π∼ Dir(20.49, 21.22, 20.46, 20.97) and q∼ Dir(7.72, 3.16, 3.24, 3.18, 2.69, 7.45). Other parameters were the same as for tree ii.

### Likelihood Ratio Test of Gene Flow and ML Estimation of Migration Rates

Since bpp currently does not allow gene flow between species, we performed a separate analysis using an IM model implemented in the ML program 3s (Zhu and Yang 2012; Dalquen et al. 2017). The implementation works with three species only (*S*_1_, *S*_2_, and *S*_3_) assuming the species tree ((*S*_1_, *S*_2_), *S*_3_), and only allows gene flow between the two ingroup species (*S*_1_, *S*_2_) with migration rates *M*_12_ and $M_{21}$, while species *S*_3_ is used as an outgroup. Here, $M_{ij}=N_{j}m_{ij}$ is the expected number of individuals migrating from species *i* to *j* per generation. We tested for gene flow between *A. gambiae* and *A. arabiensis*, and between *A. merus* and *A. quadriannulatus*, as suggested by conflicting species tree estimates from the bpp analysis. We used *A. christyi* (O) as the outgroup (species *S*_3_). Thus we analyzed two species triplets, GAO and RQO, with a fixed species tree ((GA)O) and ((RQ)O), respectively. As *A. christyi* is a very distant outgroup, we also analyzed the GAL, GAR, and RQL triplets, using *A. melas* or *A. merus* as the outgroup. The noncoding loci were used, and different chromosomal arms and inversion regions were analyzed separately, as well as the entire chromosome arms and all autosomal regions as a whole. For each locus, we chose one of the following three locus configurations uniformly at random: 123, 113, and 223. Here, 123 means 1 sequence from each of the three species, and 113 means 2 sequences from species *S*_1_ and 1 sequence from species *S*_3_, etc. When one sequence from a species was used, it was always from the reference genome. We estimated parameters under two models, M0 (no gene flow) and M2 (gene flow), and compared them using a likelihood ratio test (LRT). Model M0 has two species divergence times (*τ*_0_ for the root, and *τ*_1_ for the two ingroup species) and four effective population sizes: $\theta_{1},\theta_{2},\theta_{4},\theta_{5}$ (for the two extant populations, *S*_1_ and *S*_2_, and for two ancestral populations, *S*_4_ for the root and *S*_5_ for the ingroup species). There is no population size parameter for the outgroup (*θ*_3_) since we always used one outgroup sequence. Model M2 has two additional parameters *M*_12_ and *M*_21_. Integration over the two coalescent times in the gene trees in the likelihood calculation used Gaussian quadrature with 32 points (Yang 2002). We ran the program twice for each analysis, and the run with a higher log-likelihood value was used.

## Supplementary Material

Supplementary Data
