# Supplementary material for: Coalescent Analysis of Phylogenomic Data Confidently Resolves the Species Relationships in the Anopheles gambiae Species Complex
Source: Mol Biol Evol. 2018 Aug 9;35(10):2512–27. doi: 10.1093/molbev/msy158 (PMC6188554; doi:10.1093/molbev/msy158)
Supplement: Supplementary Data [file msy158_supp.zip › anopheles.SI.pdf]

Supplementary material

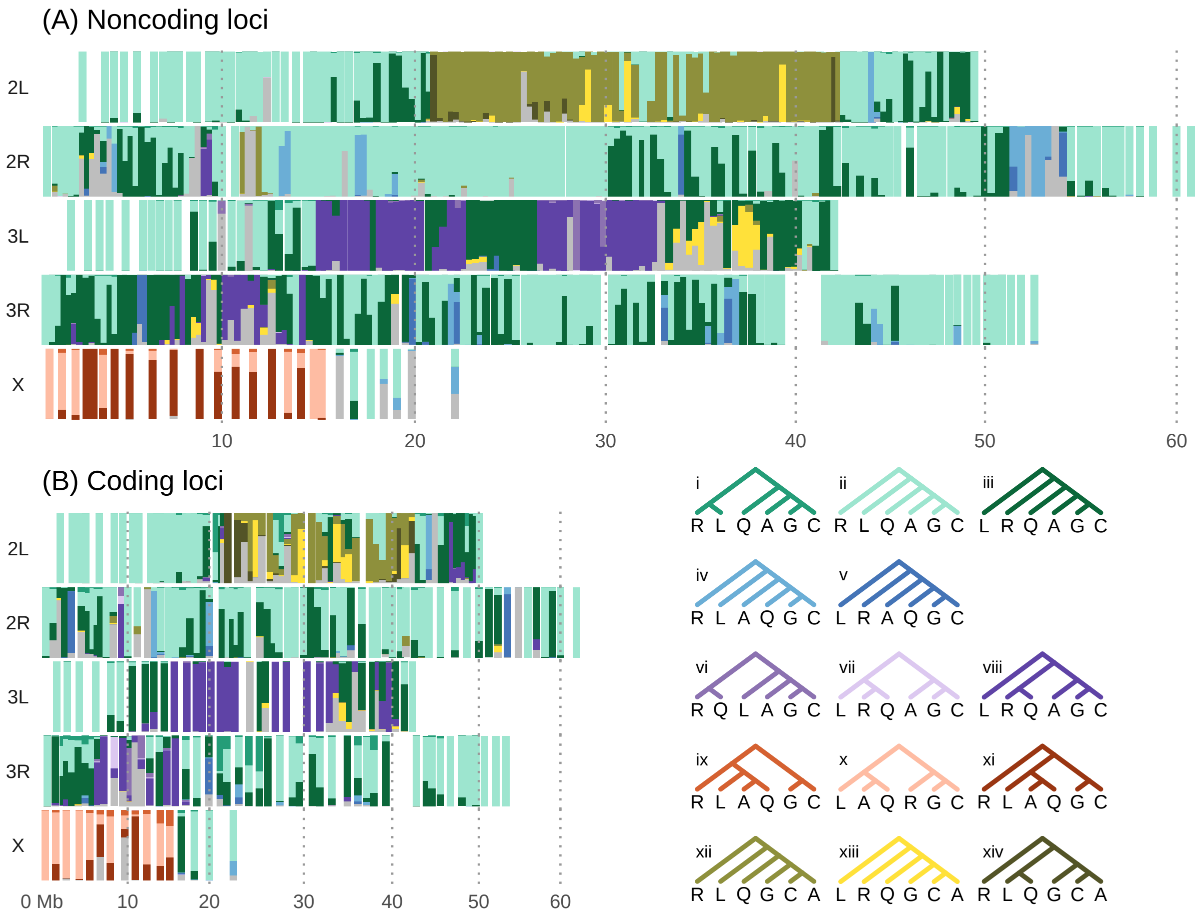

Figure S1: Posterior probabilities of species trees inferred under the MSC model using BPP when the outgroup species *A. christyi* is included. The outgroup is always the earliest branching species in the MAP trees and is omitted in the tree diagrams. See legend to fig. 1.

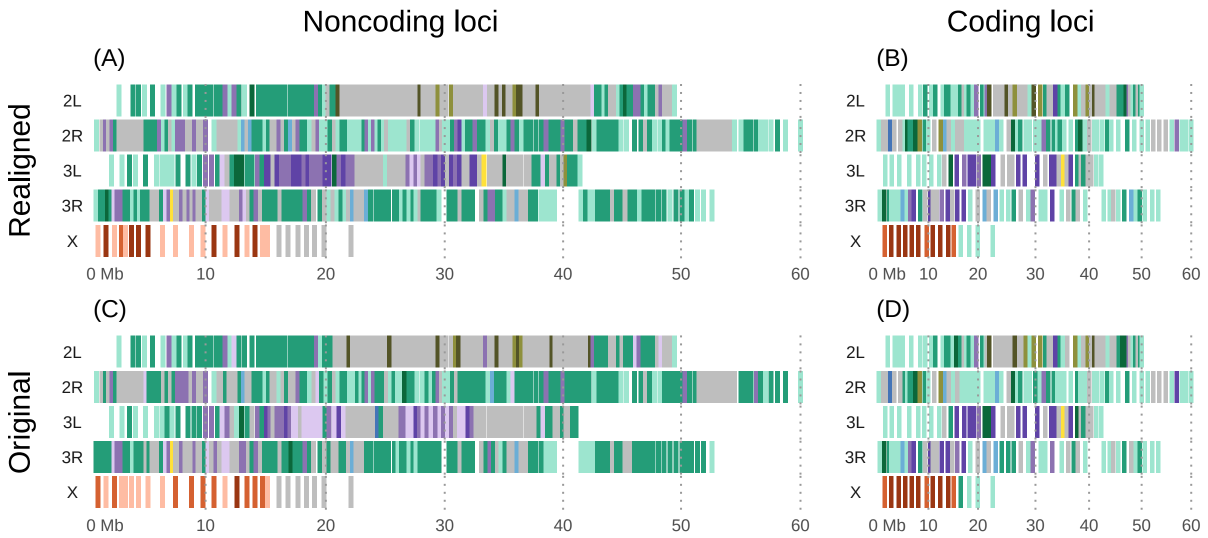

Figure S2: ML concatenation trees inferred using RAxML from blocks of 100 loci. The reference genome is used for each ingroup species, and the results for the non-reference genomes are virtually identical and not shown. Trees are defined in fig. 1.

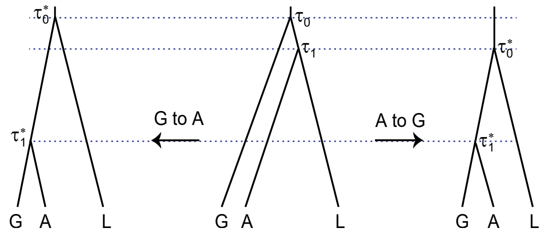

Figure S3: Introgression changes species relationships and reduces divergence times (Fontaine *et al.*, 2015, fig. S16). For the GAL triplet, A-to-G introgression leads to the tree ((GA)L), with divergence times $\tau_0^* = \tau_1$ and $\tau_1^* < \tau_1$, while G-to-A introgression leads to the tree ((GA)L), with $\tau_0^* = \tau_0$ and $\tau_1^* < \tau_1$.

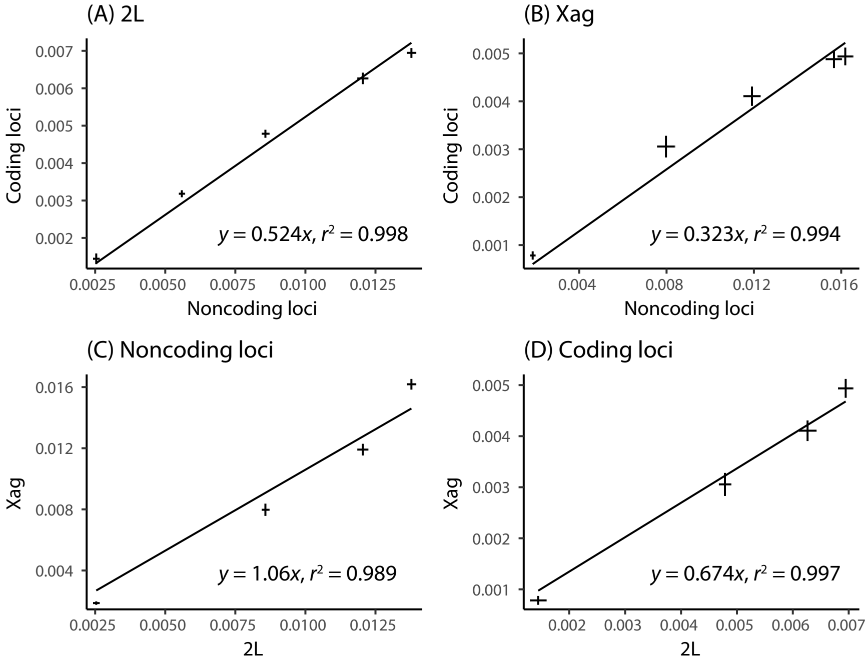

Figure S4: Posterior means of species divergence times (τ) from different datasets (see fig. 2). The bars represent the 95% CIs.

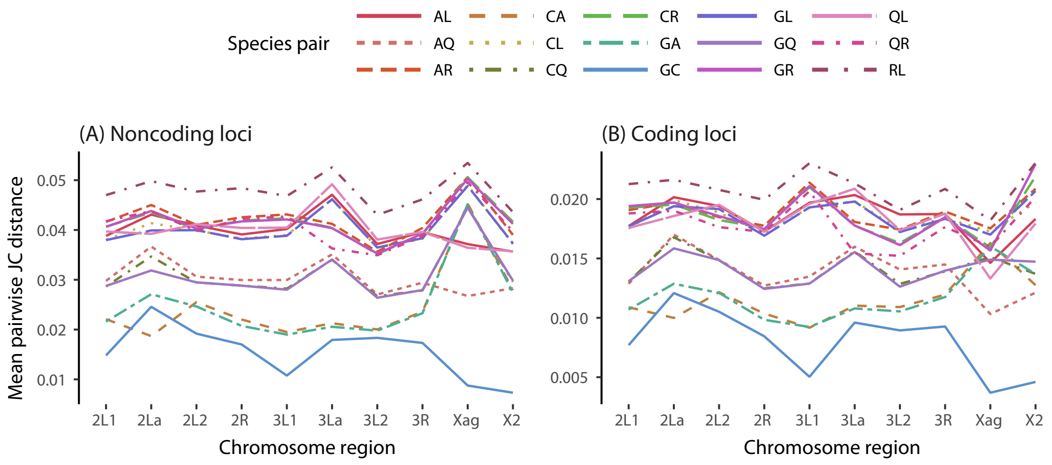

Figure S5: Pairwise JC distance from the whole-genome data, averaged over loci for each chromosomal region.

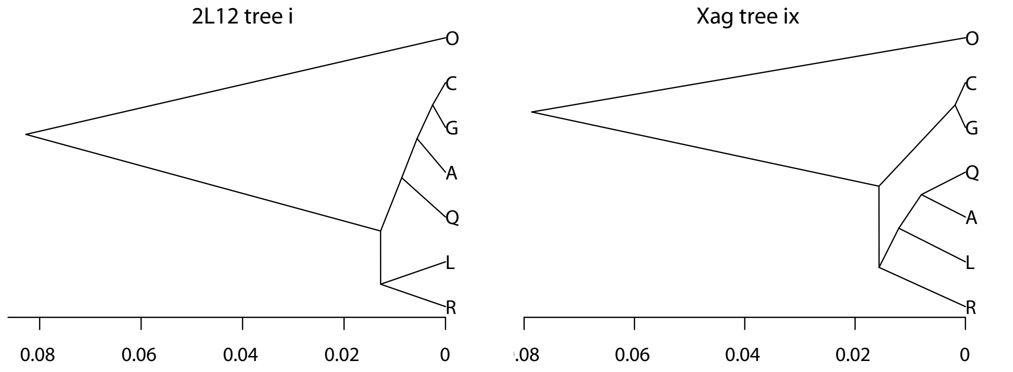

Figure S6: Species trees i and ix with BPP estimates of species divergence times (τs). The parameter estimates were used to simulate data to be analyzed using concatenation and BPP.

Table S1: Number of loci in each chromosome region in noncoding and coding datasets.

Dataset	Chromosome region										Total
	2L1	2La	2L2	2R	3L1	3La	3L2	3R	Xag	X2	
Noncoding	4134	6732	2330	17027	2496	6280	1823	14323	1825	622	57592
Coding	2223	2776	1362	6849	983	1998	764	4977	1179	394	23505

Table S2: Proportions of inferred species trees (with the minimum, median and maximum support values for the inferred tree in parentheses) for noncoding and coding loci from BPP and RAxML by chromosomal regions

Tree	Noncoding loci			Coding loci		
	BPP	RAxML (reference)	RAxML (non-reference)	BPP	RAxML (reference)	RAxML (non-reference)
			**Autosomes (excluding 2La and 3La)**			
i	0	0.4118 (0.32, 0.76, 0.99)	0.4965 (0.25, 0.73, 1.00)	0.0057 (0.42, 0.42, 0.42)	0.1667 (0.35, 0.63, 0.91)	0.2069 (0.25, 0.57, 0.87)
ii	0.5882 (0.48, 1.00, 1.00)	0.2188 (0.33, 0.69, 1.00)	0.2400 (0.41, 0.73, 1.00)	0.4655 (0.43, 0.97, 1.00)	0.4655 (0.26, 0.77, 1.00)	0.5000 (0.36, 0.79, 1.00)
iii	0.2776 (0.31, 0.92, 1.00)	0.0141 (0.44, 0.62, 0.73)	0.0282 (0.32, 0.58, 0.77)	0.3678 (0.39, 0.82, 1.00)	0.0402 (0.52, 0.62, 0.72)	0.0632 (0.39, 0.58, 0.85)
iv	0.0306 (0.49, 0.94, 1.00)	0.0141 (0.62, 0.71, 0.91)	0	0.0115 (0.82, 0.89, 0.95)	0.0345 (0.43, 0.63, 0.77)	0
v	0.0188 (0.54, 0.80, 0.99)	0	0	0.0230 (0.38, 0.69, 0.99)	0.0057 (0.56, 0.56, 0.56)	0
vi	0	0.0965 (0.34, 0.57, 0.99)	0.1388 (0.31, 0.64, 1.00)	0.0115 (0.44, 0.60, 0.76)	0.0460 (0.40, 0.60, 0.89)	0.0632 (0.30, 0.44, 0.92)
vii	0	0.0212 (0.44, 0.62, 0.88)	0.0259 (0.29, 0.57, 0.83)	0	0	0
viii	0.0353 (0.38, 0.76, 1.00)	0.0024 (0.28, 0.28, 0.28)	0.0094 (0.38, 0.49, 0.68)	0.0690 (0.35, 0.93, 0.97)	0.0402 (0.27, 0.48, 0.69)	0.0690 (0.32, 0.61, 0.85)
ix	0	0	0	0	0	0
x	0	0	0	0	0	0
xi	0	0	0	0	0	0
xii	0.0047 (0.89, 0.94, 1.00)	0.0024 (0.45, 0.45, 0.45)	0	0	0.0115 (0.55, 0.63, 0.71)	0
xiii	0.0094 (0.40, 0.56, 0.91)	0.0024 (0.58, 0.58, 0.58)	0	0	0	0.0057 (0.79, 0.79, 0.79)
xiv	0	0	0	0	0	0
			**2La**			
i	0	0	0	0.0357 (0.77, 0.77, 0.77)	0.1071 (0.43, 0.63, 0.72)	0
ii	0.0588 (0.53, 0.81, 0.99)	0	0	0.1071 (0.29, 0.44, 0.77)	0.1071 (0.62, 0.66, 0.69)	0
iii	0.0147 (0.53, 0.53, 0.53)	0	0	0.0357 (0.66, 0.66, 0.66)	0	0
iv	0	0	0	0	0	0
v	0	0	0	0	0	0
vi	0	0	0	0	0	0
vii	0	0.0147 (0.75, 0.75, 0.75)	0	0	0	0
viii	0	0	0	0	0.0357 (0.76, 0.76, 0.76)	0
ix	0	0	0	0	0	0
x	0	0	0	0	0	0
xi	0	0	0	0	0	0
xii	0.8088 (0.51, 0.97, 1.00)	0.0441 (0.47, 0.49, 0.67)	0.1471 (0.28, 0.60, 0.85)	0.4643 (0.15, 0.71, 1.00)	0.1429 (0.28, 0.47, 0.72)	0.3571 (0.41, 0.69, 0.91)
xiii	0.0588 (0.57, 0.83, 0.98)	0	0.0147 (0.29, 0.29, 0.29)	0.1429 (0.43, 0.55, 0.99)	0	0.0357 (0.49, 0.49, 0.49)
xiv	0.0441 (0.55, 0.92, 0.94)	0.1029 (0.36, 0.60, 0.86)	0.0735 (0.24, 0.65, 0.76)	0.1429 (0.63, 0.79, 1.00)	0.1429 (0.35, 0.50, 0.54)	0.1071 (0.46, 0.59, 0.61)

Table S2: Continued.

Tree	Noncoding loci			Coding loci		
	BPP	RAxML (reference)	RAxML (non-reference)	BPP	RAxML (reference)	RAxML (non-reference)
			**3La**			
i	0	0	0.0164 (0.78, 0.78, 0.78)	0	0	0
ii	0	0.0164 (0.33, 0.33, 0.33)	0.0164 (0.84, 0.84, 0.84)	0	0	0
iii	0.3540 (0.36, 0.99, 1.00)	0.0328 (0.50, 0.62, 0.73)	0.0164 (0.79, 0.79, 0.79)	0.2000 (0.62, 0.76, 1.00)	0.1000 (0.66, 0.69, 0.72)	0.0500 (0.78, 0.78, 0.78)
iv	0	0	0	0	0	0
v	0.0159 (0.77, 0.77, 0.77)	0	0	0	0	0
vi	0.0476 (0.70, 0.97, 1.00)	0.2787 (0.35, 0.68, 0.86)	0.3279 (0.49, 0.78, 0.92)	0	0.1000 (0.83, 0.87, 0.90)	0.1500 (0.60, 0.62, 0.91)
vii	0	0.0328 (0.52, 0.53, 0.54)	0.1148 (0.47, 0.71, 0.91)	0	0	0
viii	0.5714 (0.69, 1.00, 1.00)	0.2623 (0.40, 0.59, 0.75)	0.4754 (0.47, 0.75, 0.95)	0.7000 (0.51, 1.00, 1.00)	0.4500 (0.56, 0.66, 0.93)	0.6000 (0.54, 0.85, 0.98)
ix	0	0	0	0	0	0
x	0	0	0	0	0	0
xi	0	0	0	0	0	0
xii	0	0	0	0	0	0
xiii	0.0159 (0.54, 0.54, 0.54)	0.0164 (0.69, 0.69, 0.69)	0	0	0.0500 (0.70, 0.70, 0.70)	0
xiv	0	0	0	0	0	0
			**Xag**			
i	0	0	0	0	0	0
ii	0	0	0	0	0	0
iii	0	0	0	0	0	0
iv	0	0	0	0	0	0
v	0	0	0	0	0	0
vi	0	0	0	0	0	0
vii	0	0	0	0	0	0
viii	0	0	0	0	0	0
ix	0	0.0526 (0.51, 0.51, 0.51)	0.1053 (0.57, 0.61, 0.65)	0.0833 (0.73, 0.73, 0.73)	0.2500 (0.46, 0.48, 0.65)	0.1667 (0.50, 0.55, 0.59)
x	0.3684 (0.83, 0.92, 1.00)	0.5789 (0.45, 0.69, 0.97)	0.5263 (0.38, 0.62, 0.95)	0.5833 (0.57, 0.97, 1.00)	0.0833 (0.97, 0.97, 0.97)	0.0833 (0.93, 0.93, 0.93)
xi	0.6316 (0.43, 0.99, 1.00)	0.3684 (0.53, 0.91, 0.94)	0.3684 (0.65, 0.78, 0.94)	0.1667 (0.35, 0.52, 0.69)	0.6667 (0.56, 0.74, 1.00)	0.7500 (0.58, 0.71, 0.99)
xii	0	0	0	0	0	0
xiii	0	0	0	0	0	0
xiv	0	0	0	0	0	0

Note.—See legend to table 2.

Table S3: MLEs ($\times 10^{-2}$) from 3S analysis of triplet data under models M0 (no gene flow) and M2 (with gene flow)

Chr	Model	τ_1	τ_0	θ_4	θ_5	θ_1	θ_2	M_{12}	M_{21}	$2\Delta\ell$
				**GAO, species tree ((GA)O)**						
2L12	M0	0.47	7.28	11.38	0.93	2.93	0.99			
	M2	0.50	7.27	11.38	0.92	2.63	0.98	0.00	11.13	3.70
2La	M0	0.50	8.28	10.53	1.17	49.41	1.04			
	M2	0.50	8.28	10.53	1.17	52.04	1.03	0.00	20.69	2.21
2L	M0	0.47	7.69	11.12	1.07	6.24	1.01			
	M2	0.56	7.69	11.12	1.04	4.16	0.98	0.00	60.12	43.10
2R	M0	0.42	7.55	11.29	0.86	3.67	1.50			
	M2	0.42	7.55	11.29	0.86	3.67	1.50	0.00	0.00	0.00
3L12	M0	0.37	7.82	10.44	0.90	1.79	1.14			
	M2	0.40	7.82	10.44	0.88	1.58	1.13	0.00	12.14	3.79
3La	M0	0.47	8.12	9.51	0.82	10.18	1.80			
	M2	0.47	8.12	9.51	0.82	9.49	1.79	0.00	12.63	0.21
3L	M0	0.42	7.97	9.95	0.87	3.40	1.44			
	M2	0.46	7.97	9.96	0.84	2.73	1.39	0.00	29.39	13.56
3R	M0	0.47	7.39	10.81	0.98	3.34	1.67			
	M2	0.47	7.39	10.81	0.98	3.34	1.67	0.00	0.00	0.00
auto	M0	0.43	7.51	11.00	0.92	3.17	1.42			
	M2	0.44	7.51	11.00	0.92	3.12	1.42	0.00	2.08	1.10
Xag	M0	1.08	7.44	13.62	1.74	0.71	0.34			
	M2	1.12	7.44	13.63	1.70	0.71	0.33	0.17	0.00	1.39
X2	M0	0.75	9.32	10.97	0.95	0.35	0.38			
	M2	0.75	9.32	10.97	0.95	0.35	0.38	0.00	0.00	0.00
				**GAR, species tree ((GA)R)**						
2L12	M0	0.64	1.21	1.49	0.62	2.97	1.11			
	M2	0.74	1.20	1.51	0.51	2.36	1.07	0.00	17.07	30.13
2La	M0	0.64	1.37	1.50	0.88	32.18	1.11			
	M2	1.28	1.34	1.56	0.10	12.76	1.01	0.00	365.67	197.87
2L	M0	0.62	1.28	1.51	0.77	6.30	1.11			
	M2	0.85	1.26	1.54	0.52	3.76	1.05	0.00	60.43	146.54
2R	M0	0.59	1.22	1.53	0.55	3.92	1.58			
	M2	0.61	1.21	1.54	0.53	3.55	1.55	0.00	11.68	9.20
3L12	M0	0.46	1.12	1.55	0.73	2.12	1.06			
	M2	0.61	1.11	1.58	0.56	1.49	1.03	0.00	25.85	26.47
3La	M0	0.60	1.25	1.40	0.60	13.39	1.91			
	M2	0.67	1.25	1.41	0.54	8.55	1.80	0.00	90.19	9.95
3L	M0	0.54	1.19	1.46	0.66	4.23	1.51			
	M2	0.72	1.18	1.49	0.47	2.57	1.39	0.00	53.68	78.85
3R	M0	0.70	1.12	1.55	0.50	3.78	1.81			
	M2	0.81	1.11	1.57	0.36	2.89	1.72	0.00	24.74	59.11
auto	M0	0.61	1.17	1.54	0.58	3.43	1.49			
	M2	0.69	1.16	1.56	0.49	2.70	1.43	0.00	21.93	124.48
Xag	M0	1.37	1.37	1.92	0.01	0.77	0.37			
	M2	1.37	1.37	1.91	0.01	0.77	0.37	0.00	0.05	0.90
X2	M0	0.81	1.19	1.50	0.58	0.37	0.36			
	M2	0.81	1.19	1.50	0.58	0.37	0.36	0.00	0.00	0.00

Table S3: Continued.

Chr	Model	τ_1	τ_0	θ_4	θ_5	θ_1	θ_2	M_{12}	M_{21}	$2\Delta\ell$
				**GAL, species tree ((GA)L)**						
2L12	M0	0.59	1.16	1.40	0.66	3.49	1.17			
	M2	0.74	1.15	1.43	0.48	2.48	1.11	0.00	25.80	40.54
2La	M0	0.65	1.31	1.38	0.91	44.09	1.08			
	M2	1.29	1.29	1.42	0.00	16.42	1.00	0.00	467.54	175.21
2L	M0	0.60	1.22	1.41	0.81	7.70	1.13			
	M2	0.89	1.21	1.45	0.45	4.03	1.06	0.00	80.37	154.76
2R	M0	0.60	1.15	1.38	0.54	3.94	1.58			
	M2	0.64	1.15	1.38	0.49	3.35	1.53	0.00	18.46	19.36
3L12	M0	0.50	1.13	1.52	0.65	2.10	1.25			
	M2	0.77	1.10	1.58	0.33	1.29	1.11	2.27	30.75	61.61
3La	M0	0.59	1.36	1.63	0.62	11.80	2.12			
	M2	0.65	1.36	1.64	0.57	7.63	1.97	0.00	99.92	14.16
3L	M0	0.57	1.23	1.62	0.61	3.78	1.63			
	M2	0.75	1.21	1.66	0.42	2.26	1.46	0.00	52.54	107.60
3R	M0	0.70	1.14	1.43	0.50	3.85	1.89			
	M2	0.75	1.13	1.45	0.42	3.21	1.82	0.00	18.53	29.59
auto	M0	0.62	1.15	1.41	0.57	3.57	1.57			
	M2	0.70	1.14	1.43	0.47	2.77	1.50	0.00	24.38	138.59
Xag	M0	1.13	1.14	1.80	1.87	0.72	0.37			
	M2	1.13	1.14	1.80	21.74	0.72	0.37	0.00	0.00	0.09
X2	M0	0.96	1.10	1.31	0.27	0.35	0.44			
	M2	1.06	1.09	1.32	0.06	0.34	0.43	0.00	0.25	4.20
				**RQO, species tree ((RQ)O)**						
2L12	M0	1.13	7.38	11.49	1.35	0.61	1.09			
	M2	1.14	7.38	11.49	1.34	0.60	1.09	0.00	0.09	0.56
2La	M0	1.30	8.35	10.60	1.28	0.76	1.22			
	M2	1.30	8.35	10.60	1.28	0.76	1.22	0.00	0.00	0.00
2L	M0	1.20	7.78	11.21	1.32	0.68	1.15			
	M2	1.21	7.78	11.21	1.32	0.68	1.15	0.00	0.06	0.48
2R	M0	1.12	7.63	11.47	1.39	0.60	1.24			
	M2	1.13	7.63	11.48	1.39	0.60	1.23	0.11	0.00	0.88
3L12	M0	1.02	7.88	10.61	1.50	0.63	0.87			
	M2	1.06	7.88	10.61	1.47	0.61	0.87	0.00	0.44	4.20
3La	M0	1.07	8.22	9.64	0.97	0.94	1.83			
	M2	1.08	8.22	9.64	0.96	0.94	1.81	0.45	0.00	1.53
3L	M0	1.04	8.05	10.11	1.21	0.79	1.30			
	M2	1.07	8.05	10.11	1.20	0.79	1.28	0.59	0.00	5.67
3R	M0	1.02	7.46	10.94	1.29	0.70	1.45			
	M2	1.03	7.46	10.94	1.28	0.70	1.44	0.17	0.00	0.64
auto	M0	1.08	7.58	11.15	1.36	0.64	1.24			
	M2	1.09	7.58	11.15	1.36	0.64	1.23	0.18	0.00	3.79
Xag	M0	1.15	7.47	13.78	2.01	0.48	0.53			
	M2	1.38	7.46	13.81	1.77	0.46	0.54	0.00	0.67	14.48
X2	M0	1.07	9.25	11.47	1.51	0.20	0.26			
	M2	1.08	9.25	11.47	1.51	0.20	0.26	0.00	0.00	0.00

Table S3: Continued.

Chr	Model	τ_1	τ_0	θ_4	θ_5	θ_1	θ_2	M_{12}	M_{21}	$2\Delta\ell$
**RQL, species tree ((RQ)L)**										
2L12	M0	1.30	1.30	1.57	0.01	0.62	1.20			
	M2	1.30	1.30	1.57	0.01	0.62	1.20	0.00	0.00	0.00
2La	M0	1.38	1.38	1.49	0.03	0.76	1.40			
	M2	1.38	1.38	1.49	0.03	0.76	1.40	0.00	0.00	0.00
2L	M0	1.34	1.34	1.54	0.02	0.69	1.29			
	M2	1.34	1.34	1.54	0.02	0.69	1.29	0.00	0.06	0.00
2R	M0	1.29	1.29	1.62	0.02	0.62	1.32			
	M2	1.29	1.29	1.62	0.02	0.62	1.32	0.00	0.00	0.00
3L12	M0	1.22	1.22	1.61	0.01	0.63	0.92			
	M2	1.22	1.22	1.61	0.01	0.63	0.92	0.00	0.00	0.00
3La	M0	1.52	1.52	1.64	0.00	1.01	1.87			
	M2	1.51	1.51	1.79	0.00	1.02	1.79	0.67	0.00	44.40
3L	M0	1.37	1.37	1.76	0.00	0.84	1.40			
	M2	1.38	1.38	1.75	0.00	0.84	1.38	0.12	0.00	2.59
3R	M0	1.24	1.25	1.62	0.01	0.73	1.55			
	M2	1.24	1.25	1.62	0.01	0.73	1.55	0.00	0.00	0.00
auto	M0	1.27	1.27	1.61	0.01	0.66	1.32			
	M2	1.27	1.27	1.61	0.01	0.66	1.32	0.00	0.00	0.00
Xag	M0	1.15	1.15	2.01	1.53	0.52	0.64			
	M2	1.15	1.15	2.01	56.82	0.52	0.64	0.00	0.00	0.32
X2	M0	1.18	1.18	1.50	1.01	0.20	0.28			
	M2	1.18	1.18	1.50	17.05	0.20	0.28	0.00	0.00	0.01

Note.— Chr, chromosomal regions: 2L12 = 2L1 + 2L2 = 2L without 2La, 3L12 = 3L1 + 3L2 = 3L without 3La, and auto = 2L12 + 2R + 3L12 + 3R (autosomes without 2La and 3La). The likelihood ratio test statistic ($2\Delta\ell$) for testing models M0 (no gene flow) against M2 (gene flow) is compared with the critical values 4.61 at 10% level, 5.99 at 5% level, and 9.21 at 1% level.

Table S4: Relative rates for noncoding loci in different chromosomal regions

chr	τ_1 (LRO)	θ_5(LRO)	d_{JC} (RL)
2L12	1.055	0.977	1.018
2La	1.113	0.983	1.071
2R	1.025	1.019	1.040
3L12	0.966	0.987	0.966
3La	1.116	1.077	1.130
3R	0.964	0.978	0.992
auto	1	1	1
Xag	1.015	1.260	1.149
X2	0.873	1.161	0.937

Note.— The relative rates are calculated using the MLEs of τ_1 or θ_5 in the 3S analysis of the LRO triplet data or using the JC distance between R and L, rescaled relative to the autosomes (fig. S5). While θs for modern species may be used, the data from Fontaine *et al.* (2015) are haploid consensus sequences generated from diploid samples, so that information concerning nucleotide diversity may be partially lost. Estimates based on the ancestral θ_5 may be affected by different population sizes for the autosomes and the X chromosome, while the JC distance between species may be similarly affected since it consists of one component after the species split and another component from the coalescent time in the ancestral species. Thus among the different relative-rate estimates, those based on τ_1 may be preferable.

Table S5: Proportions of inferred trees from datasets of 100 loci simulated using trees i and ix (with the minimum, median and maximum support values for the inferred tree in parentheses)

Tree	BPP	RAxML (Subset 1)	RAxML (Subset 2)
	2L data (1000 loci, 10 replicates)		
i*	0.20 (0.42, 0.64, 0.99)	0.83 (0.39, 0.84, 1.00)	0.82 (0.46, 0.86, 1.00)
ii	0.46 (0.38, 0.65, 0.99)	0.11 (0.37, 0.49, 0.97)	0.11 (0.49, 0.58, 0.94)
iii	0.34 (0.45, 0.59, 0.98)	0.06 (0.39, 0.56, 0.65)	0.05 (0.40, 0.51, 0.72)
	Xag data (1000 loci, 10 replicates)		
ix*	0.29 (0.37, 0.53, 0.94)	0.12 (0.49, 0.63, 0.91)	0.15 (0.42, 0.64, 0.88)
x	0.35 (0.35, 0.63, 0.99)	0.77 (0.40, 0.86, 1.00)	0.76 (0.49, 0.85, 1.00)
xi	0.36 (0.37, 0.58, 0.96)	0.11 (0.36, 0.66, 0.96)	0.09 (0.42, 0.64, 0.93)

Note.— Each dataset consists of 100 loci. The true species tree is either tree i or tree ix, with parameters under the MSC (θs and τs) estimated using BPP from the 2L and Xag data, respectively (fig. S6). Sequence data were simulated under the GTR+G mutation model, as in table 1. See legend to table 1.

References

Fontaine, M. C., Pease, J. B., Steele, A., Waterhouse, R. M., Neafsey, D. E., Sharakhov, I. V., Jiang, X., Hall, A. B., Catteruccia, F., Kakani, E., Mitchell, S. N., Wu, Y.-C., Smith, H. A., Love, R. R., Lawniczak, M. K., Slotman, M. A., Emrich, S. J., Hahn, M. W., and Besansky, N. J. 2015. Extensive introgression in a malaria vector species complex revealed by phylogenomics. *Science*, 347(6217): 1258524.
