# Supplementary figures and images for: Coalescent Analysis of Phylogenomic Data Confidently Resolves the Species Relationships in the Anopheles gambiae Species Complex
Source: Mol Biol Evol. 2018 Aug 9;35(10):2512–27. doi: 10.1093/molbev/msy158 (PMC6188554; doi:10.1093/molbev/msy158)

## (A) Noncoding loci

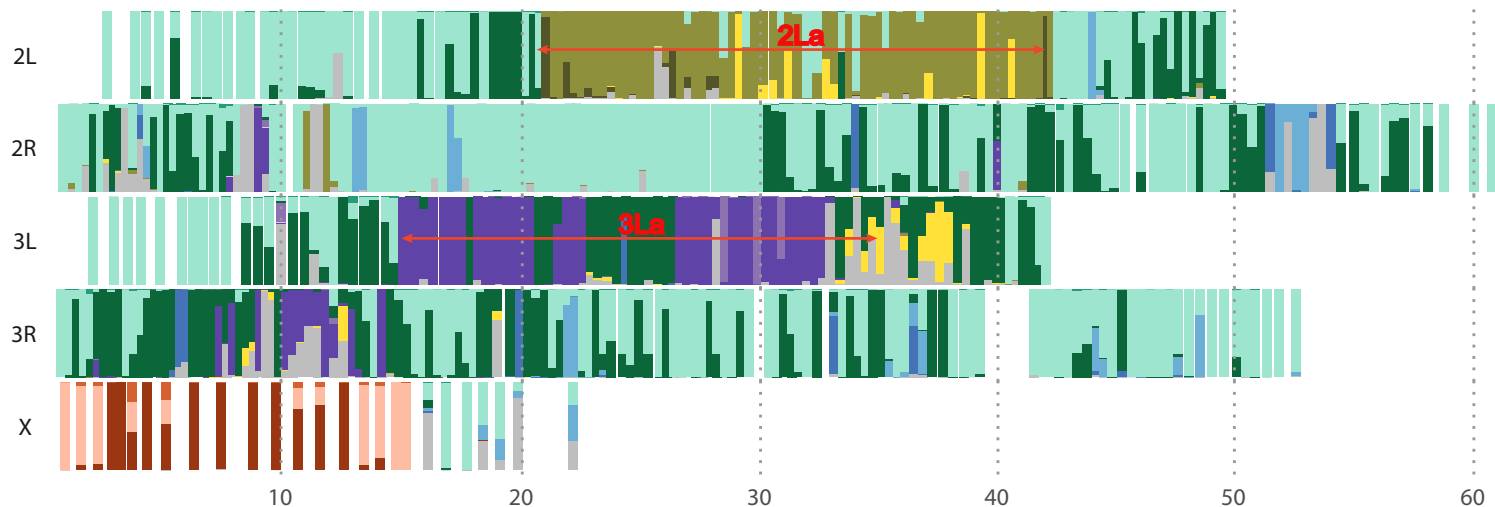

## (B) Coding loci

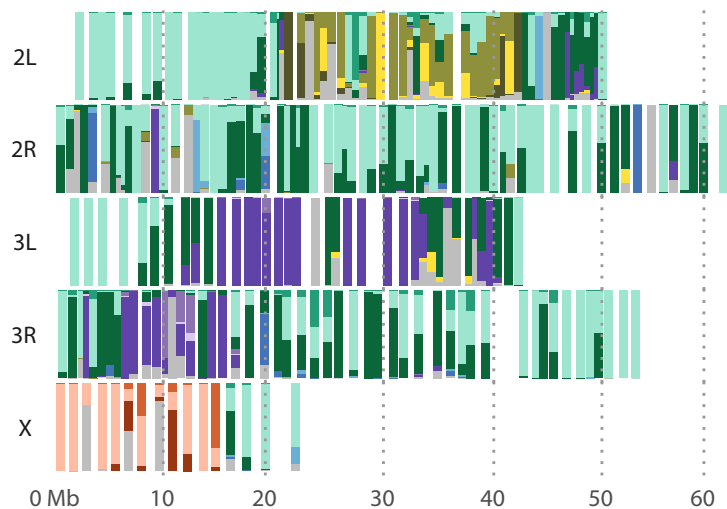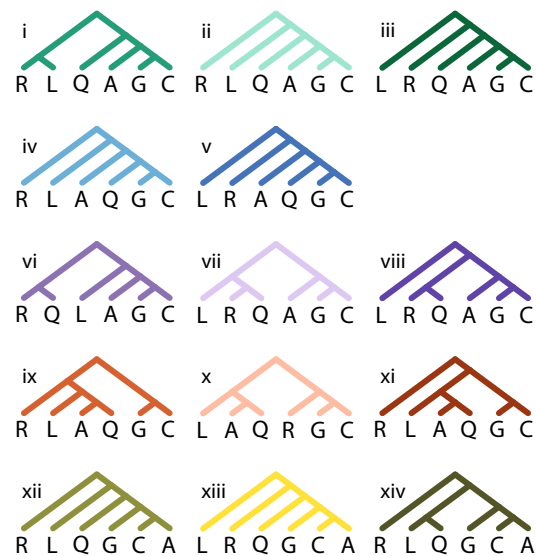

Supplement: Supplementary Data [file msy158_supp.zip › bpp-stree.pdf]

Tree ii for 2L

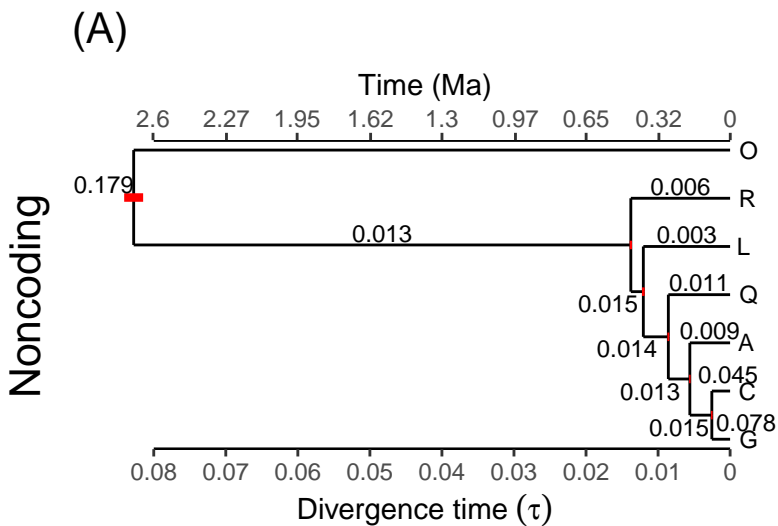

Tree xi for Xag

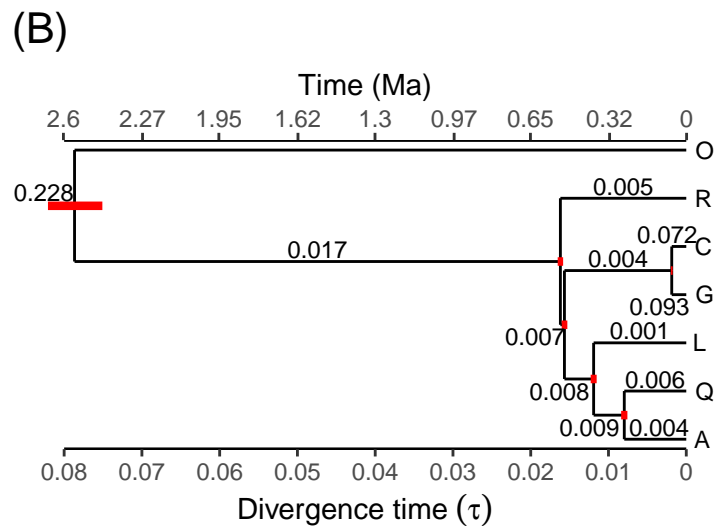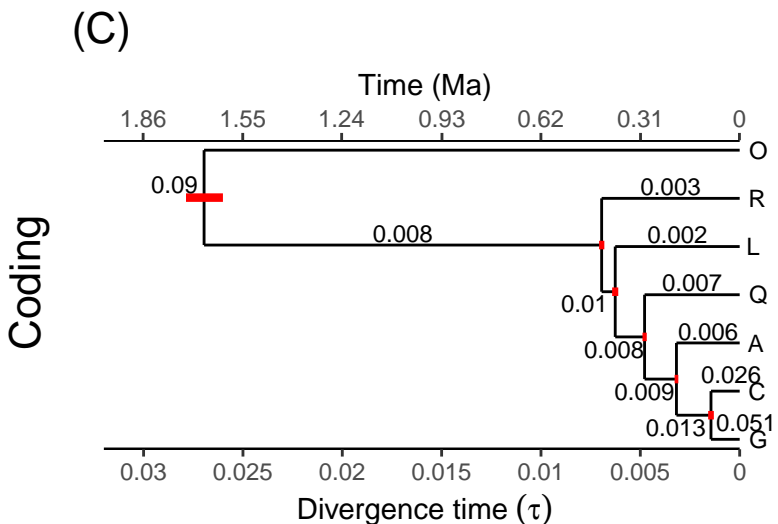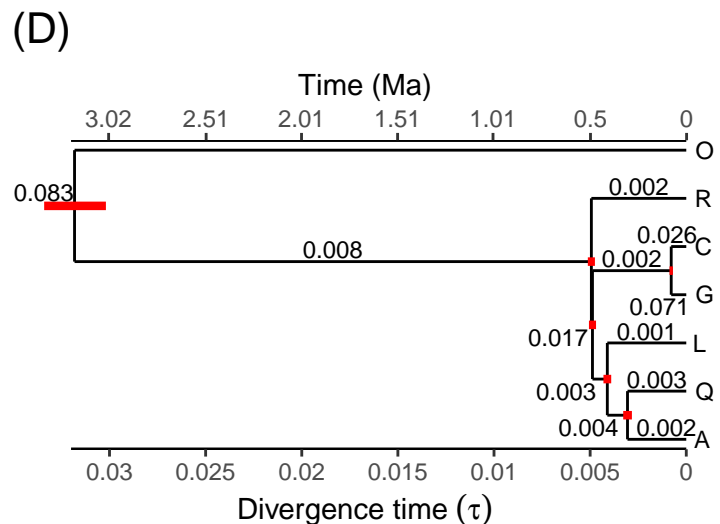

Supplement: Supplementary Data [file msy158_supp.zip › bpp-stree-A00.pdf]

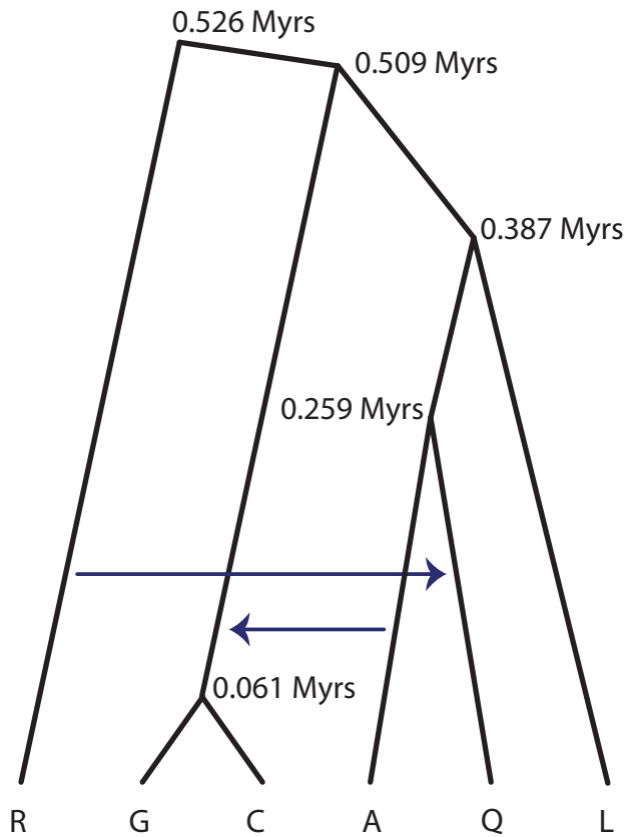

Supplement: Supplementary Data [file msy158_supp.zip › bpp-stree-summary.pdf]

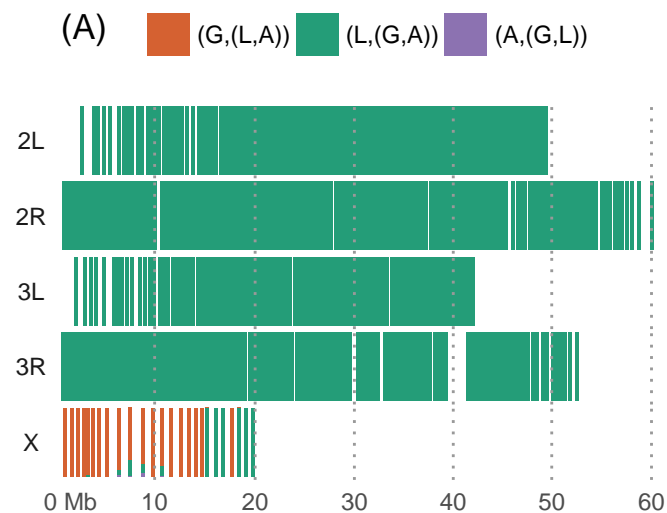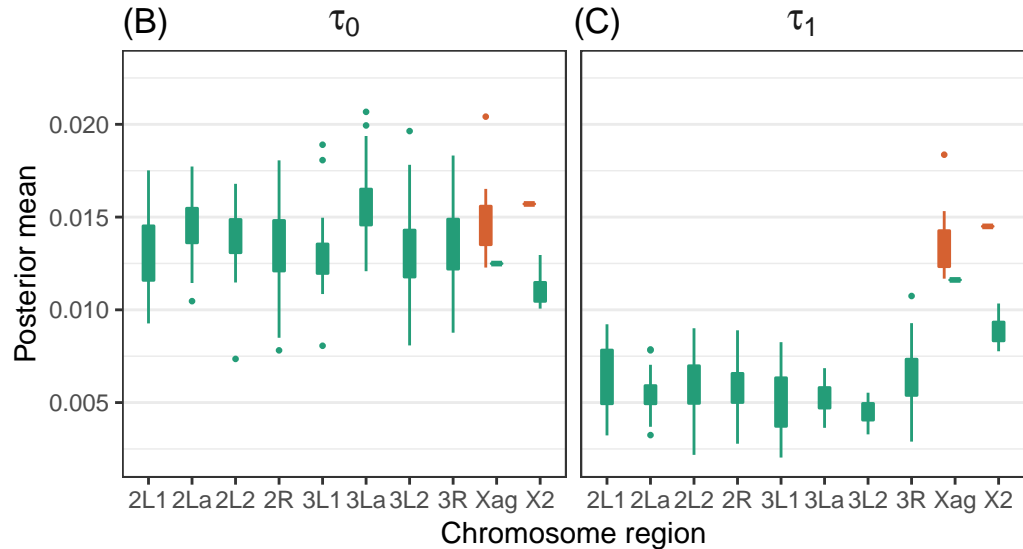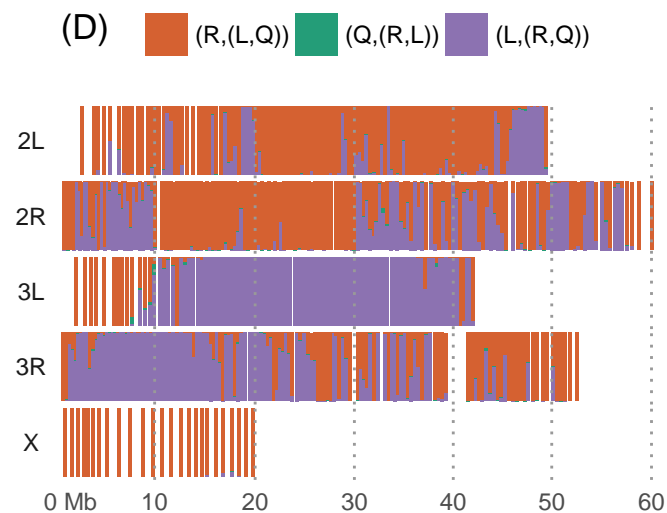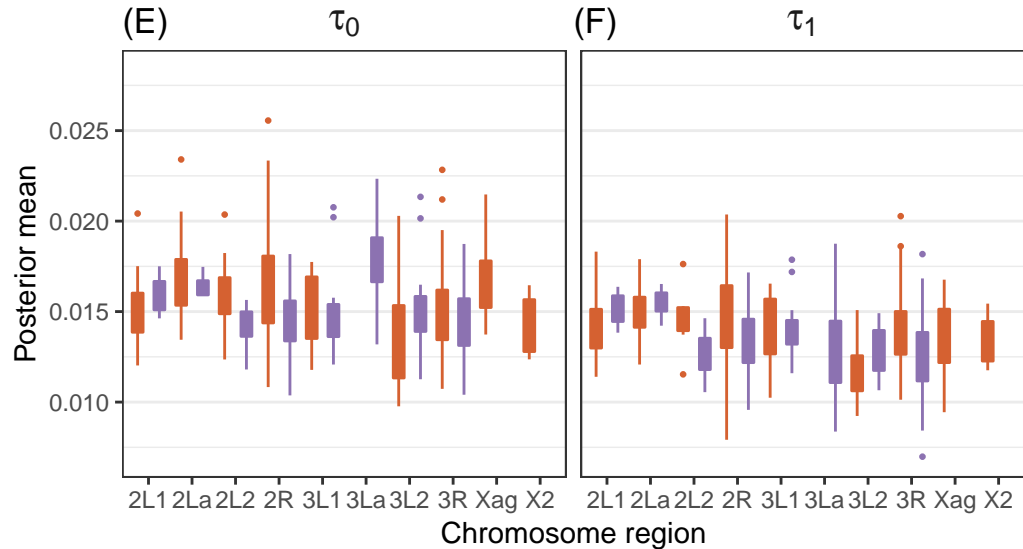

Supplement: Supplementary Data [file msy158_supp.zip › bpp-triplets.pdf]

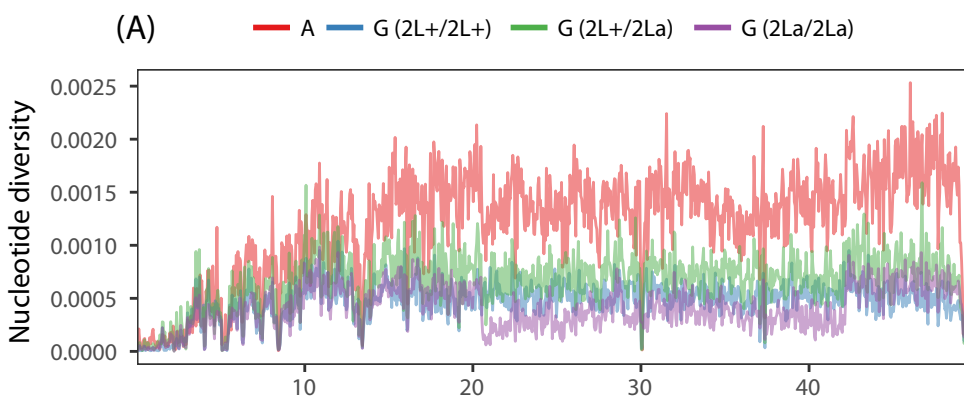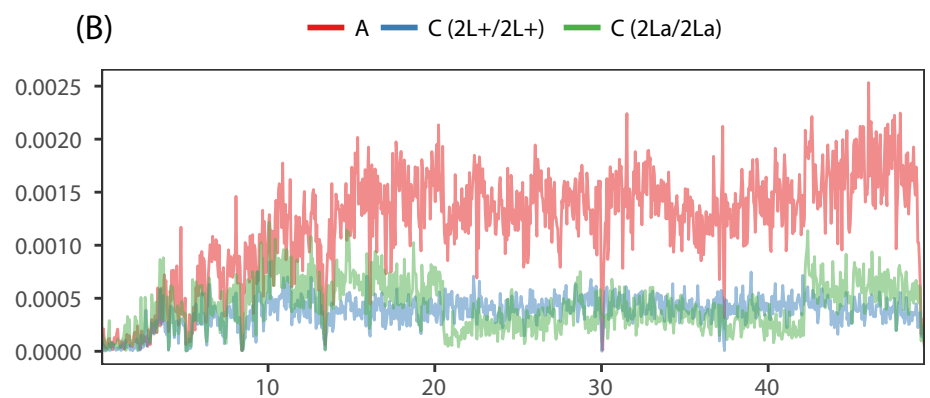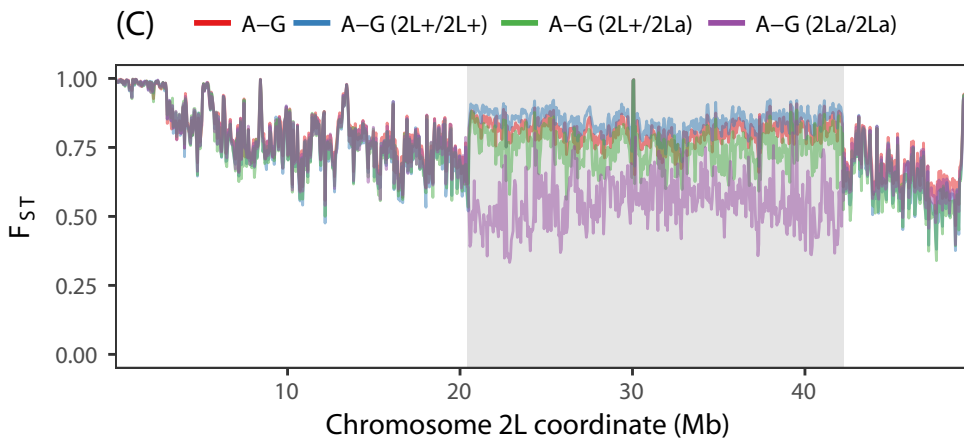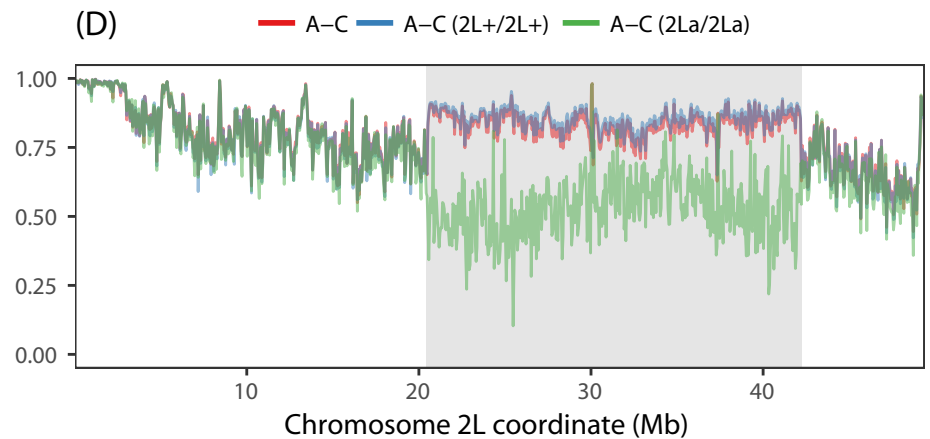

Supplement: Supplementary Data [file msy158_supp.zip › pi-fst.pdf]

(A) tree xi

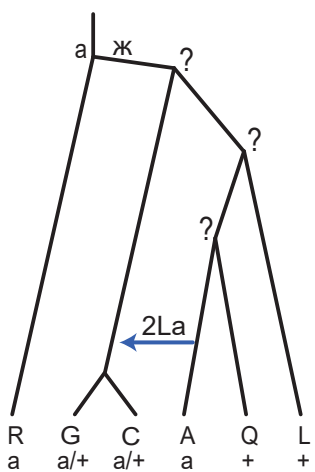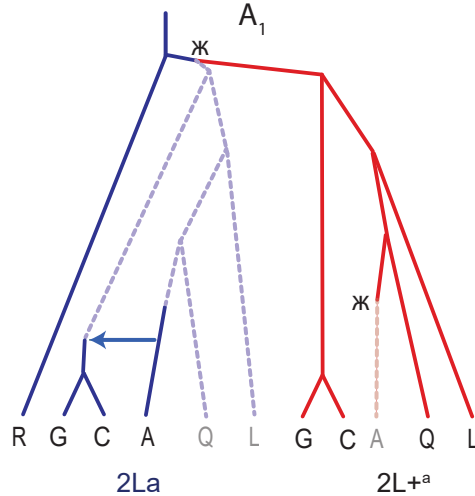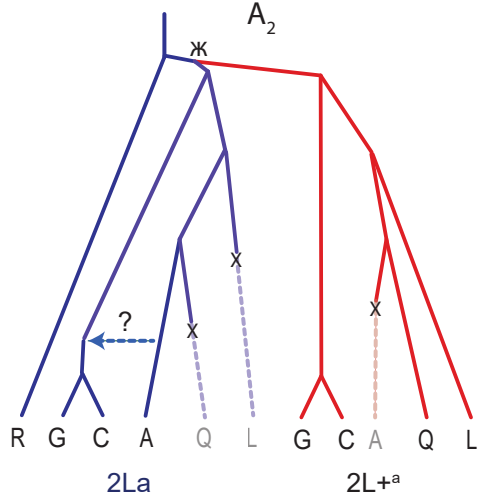

(B) tree ix

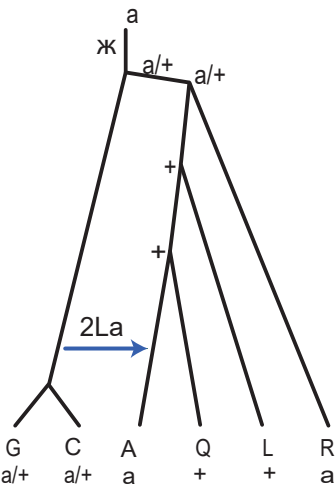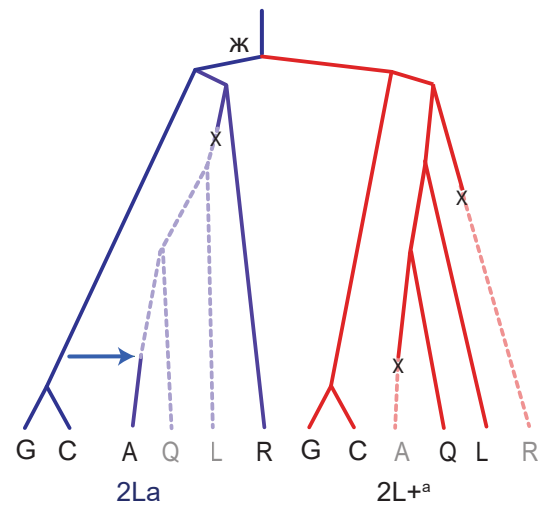

Supplement: Supplementary Data [file msy158_supp.zip › stree-2La.pdf]
